# Aminoprocalcitonin protects against hippocampal neuronal death via preserving oxidative phosphorylation in refractory status epilepticus

**DOI:** 10.1038/s41420-023-01445-7

**Published:** 2023-05-04

**Authors:** Changgeng Song, Jingjing Zhao, Jianmin Hao, Dan Mi, Jiajia Zhang, Yingying Liu, Shengxi Wu, Fang Gao, Wen Jiang

**Affiliations:** 1 Department of Neurology, Xijing Hospital, Fourth Military Medical University, 169 Chang Le Xi Road, Xi’an, 710032 Shaanxi China; 2 National Translational Science Centre for Molecular Medicine & Department of Cell Biology, Fourth Military Medical University, 169 Chang Le Xi Road, Xi’an, 710032 Shaanxi China; 3 Department of Neurobiology, Institute of Neurosciences, School of Basic Medicine, Fourth Military Medical University, 169 Chang Le Xi Road, Xi’an, 710032 Shaanxi China

**Keywords:** Epilepsy, Cell death in the nervous system

## Abstract

Refractory status epilepticus (RSE) is a neurological emergency where sustaining seizure causes severe neuronal death. Currently, there is no available neuroprotectant effective in RSE. Aminoprocalcitonin (NPCT) is a conserved peptide cleaved from procalcitonin, but its distribution and function in the brain remain enigmatic. Survival of neurons relies on sufficient energy supply. Recently, we found that NPCT was extensively distributed in the brain and had potent modulations on neuronal oxidative phosphorylation (OXPHOS), suggesting that NPCT might be involved in neuronal death by regulating energy status. In the present study, combining biochemical and histological methods, high-throughput RNA-sequence, Seahorse XFe analyser, an array of mitochondria function assays, and behavior-electroencephalogram (EEG) monitoring, we investigated the roles and translational values of NPCT in neuronal death after RSE. We found that NPCT was extensively distributed throughout gray matters in rat brain while RSE triggered NPCT overexpression in hippocampal CA3 pyramidal neurons. High-throughput RNA-sequence demonstrated that the influences of NPCT on primary hippocampal neurons were enriched in OXPHOS. Further function assays verified that NPCT facilitated ATP production, enhanced the activities of mitochondrial respiratory chain complexes I, IV, V, and increased neuronal maximal respiration capacity. NPCT exerted multiple neurotrophic effects including facilitating synaptogenesis, neuritogenesis, spinogenesis, and suppression of caspase-3. A polyclonal NPCT immunoneutralization antibody was developed to antagonize NPCT. In the in vitro 0-Mg^2+^ seizure model, immunoneutralization of NPCT caused more neuronal death, while exogenous NPCT supplementation, though did not reverse death outcomes, preserved mitochondrial membrane potential. In rat RSE model, both peripheral and intracerebroventricular immunoneutralization of NPCT exacerbated hippocampal neuronal death and peripheral immunoneutralization increased mortality. Intracerebroventricular immunoneutralization of NPCT further led to more serious hippocampal ATP depletion, and significant EEG power exhaustion. We conclude that NPCT is a neuropeptide regulating neuronal OXPHOS. During RSE, NPCT was overexpressed to protect hippocampal neuronal survival via facilitating energy supply.

## Introduction

Most seizures are brief and self-limiting [1]. Status epilepticus (SE) is a neurological emergency defined as self-sustaining seizure prolonged more than 5 min [2]. Unfortunately, between 20% and 43% of SE patients are resistant to benzodiazepines and at least one antiepileptic drug and they are referred to as refractory SE (RSE) [3]. RSE is a devastating clinical condition where sustaining drug-resistant seizure causes severe neuronal death, accompanied by a mortality over 35% [4]. Although previous studies have identified multiple mechanisms contributing to neuronal death after RSE [5], currently, there is no available neuroprotective therapy that is effective in RSE.

The brain is a highly energy-demanding organ, accounting for only 2% of the total body weight but over 20% of the resting metabolism consumption [6]. Survival of neurons relies on sufficient energy supply. Sustaining seizure activity represents a metabolic threat that pushes brain energy consumption and supply to its limits [7]. It has been demonstrated that mitochondrial dysfunction and resultant ATP depletion and energy failure determine the neuronal dying process after RSE [8]. Thus, identifying and targeting the key molecule that regulates neuronal ATP production and energy homeostasis in RSE bears the translational value to inhibit neuronal death caused by RSE.

Aminoprocalcitonin (NPCT) is the peptide cleaved from the amino terminal of procalcitonin (PCT), with the amino acid sequence of NPCT highly conserved among all vertebrates [9]. Although the parent peptide PCT is serum biomarker of infection routinely checked in clinical practices, knowledge about NPCT remains enigmatic. Tavares et al. reported that intracerebroventricular (i.c.v.) administration of human NPCT caused anorexic and catabolic behavioral consequences in rats [10–13]. Except for these, no other evidence exists concerning the biological functions of NPCT in the central nervous system (CNS).

Recently, we found that NPCT was extensively distributed throughout the brain and had potent regulations on the neuronal oxidative phosphorylation (OXPHOS). This inspires us to hypothesize that NPCT might be involved in neuronal death after RSE via regulating neuronal energy status. In the current study, we further investigated the roles and mechanisms of NPCT in neuronal death after RSE. We demonstrated that seizure-induced hippocampal NPCT overexpression was an endogenous neuroprotective mechanism to protect against neuronal death via preserving ATP supply in RSE, and exogenous NPCT supplementation bears the translational value to become a novel neuroprotectant. Our study revealed that NPCT was a novel neuropeptidergic regulator of neuronal OXPHOS, and provided a new therapeutic strategy to treat neuronal death after RSE.

## Results

### NPCT is a neuropeptide widely distributed in brain regions activated during SE

**Fig. 1:**
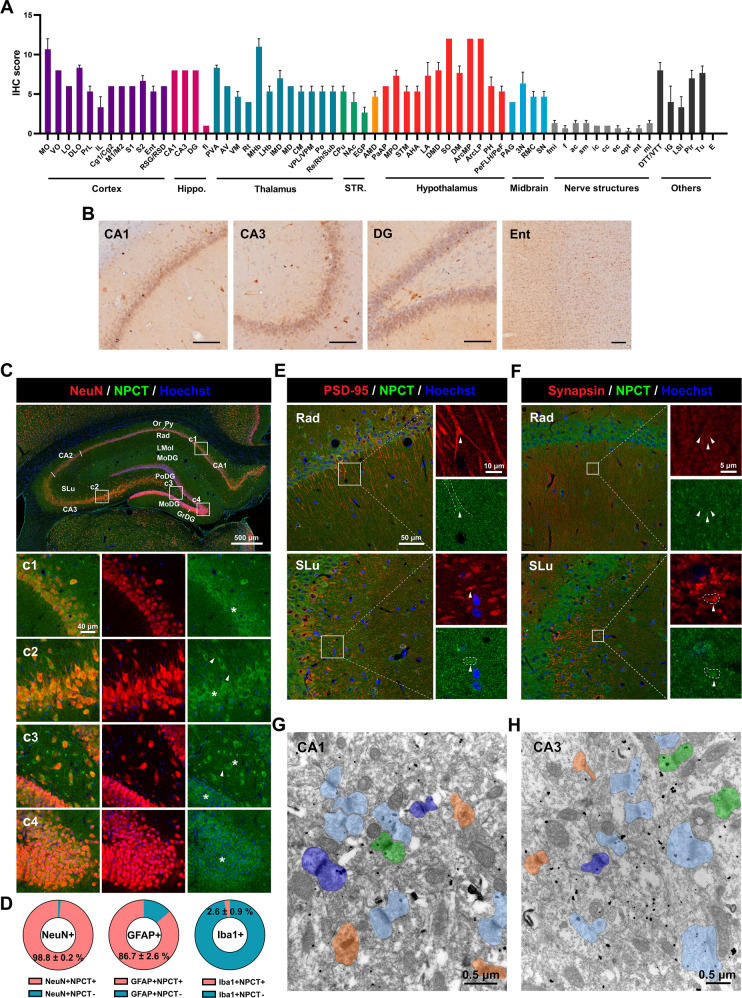
NPCT is extensively distributed throughout the gray matters and is a neuropeptide locating both presynaptically and postsynaptically in hippocampus. **A** IHC scores of the expression of NPCT in brain regions or nuclei. The higher IHC scores indicate the more intensive expression of NPCT. **B** The distribution of NPCT in brain regions activated at the initiation of SE. Bar scale = 100 μm. **C** Panorama showing the colocalization of NPCT with the neuronal marker NeuN in different territories of the rat hippocampus under physiological condition. (c1-c4) Enlarged pictures of insets from (**C**). Note that NPCT exists in neuronal somata (asterisks) and proximal dendrites (arrowheads). **D** The proportions of neurons, astrocytes, and microglia that express NPCT in the hippocampus. **E** Colocalization of NPCT and postsynaptic marker PSD-95 in the radiatum layer of CA1 and stratum lucidum of CA3. Arrowheads and dotted lines in enlarged pictures indicate the enrichment of NPCT in postsynaptic domain. **F** Colocalization of NPCT and presynaptic marker synapsin in the radiatum layer of CA1 and stratum lucidum of CA3. Arrowheads and dotted lines in enlarged pictures indicate the localization of NPCT in presynaptic domain. **G**, **H** Representative immunoelectron microscopic images showing the synaptic distribution of NPCT in CA1 (**G**) and CA3 (**H**). Synapses in (**G**, **H**) were classified into four categories based on the distribution of NPCT: NPCT in presynaptic domain (dark blue), NPCT in postsynaptic domain (light blue), NPCT in both pre- and postsynaptic domains (green), synapse without NPCT distribution (orange). Quantitative analysis demonstrated that in CA1 20.13% synapses contained NPCT in presynaptic domain, 32.89% synapses contained NPCT in postsynaptic domain, 10.74% synapses contained NPCT both pre- and postsynaptically, while 36.24% synapses had no NPCT immunoreactivity (*n* = 149 synapses from 3 rats), while the proportions in CA3 were 8.77%, 43.27%, 12.87%, and 35.09%, respectively (*n* = 171 synapses from 3 rats). All data are presented as mean ± SEM. *n* = 3 rats. CA1 field CA1 of the hippocampus, CA2 field CA2 of the hippocampus, CA3 field CA3 of the hippocampus, Ent entorhinal cortex, GrDG granular layer of the dentate gyrus, IHC immunohistochemistry, LMol lacunosum moleculare layer of the hippocampus, MoDG molecular layer of the dentate gyrus, Or oriens layer of the hippocampus, PoDG polymorph layer of the dentate gyrus, Py pyramidal cell layer of the hippocampus, Rad radiatum layer of the hippocampus, SLu stratum lucidum of the hippocampus, Other abbreviations please refer to Supplementary Fig. S1.

We started our study by investigating the overall distribution of NPCT in rat brain under physiological condition. NPCT was extensively distributed in gray matters with region specificity throughout the rat brain, and it nearly did not exist in white matters (Fig. 1A and Supplementary Fig. S1A–G). Brain regions with the highest NPCT expression (immunohistochemistry score >9 and ≤12) included the arcuate hypothalamic nucleus, the supraoptic nucleus, the medial habenular nucleus, and the medial orbital cortex (Fig. 1A). The development of SE involves a sequential activation of brain regions. At the initiation of seizure, the hippocampus and entorhinal cortex are activated [14], where NPCT was expressed in medium-high levels (Fig. 1A, B). NPCT was expressed in medium to medium-high levels in brain regions activated during the peak of SE [14], including motor, somatosensory, and piriform cortices (Fig. 1A and Supplementary Fig. S1A), motor thalamic nuclei including the anteroventral and ventromedial thalamic nuclei (Fig. 1A and Supplementary Fig. S1C), midline thalamic nuclei including the paraventricular thalamic nucleus, inter-mediodorsal, mediodorsal, rhomboid, reuniens, and submedius thalamic nuclei (Fig. 1A and Supplementary Fig. S1C), and reticular thalamic nucleus and posterior thalamic nucleus group (Fig. 1A and Supplementary Fig. S1C).

Next, we asked the cell types expressing NPCT in the hippocampus. We found that NPCT was expressed in nearly all neurons and most astrocytes in the hippocampus while its expression in microglia was neglectable (Fig. 1C, D and Supplementary Fig. S2). In neurons, NPCT was concentrated in somata and proximal dendrites (Fig. 1C), while in astrocytes NPCT mainly localized in somata rather than processes (Supplementary Fig. S2A). Moreover, NPCT was also co-localized with both PSD-95 (a postsynaptic marker) and synapsin (a presynaptic marker) in the radiatum layer of CA1 and stratum lucidum of CA3 (Fig. 1E, F), suggesting its pre- and postsynaptic existence, which was verified by immunoelectron microscopy (Fig. 1G, H). Negative control omitting the primary antibody (Supplementary Fig. S3) and antibody pre-absorbed with NPCT peptide (Supplementary Fig. S4) showed no specific staining. Collectively, these results demonstrated that NPCT was a neuropeptide locating in brain regions activated during SE, suggesting the involvement of NPCT in seizure activity.

### RSE triggered specific overexpression of NPCT in CA3 pyramidal neurons in hippocampus

**Fig. 2:**
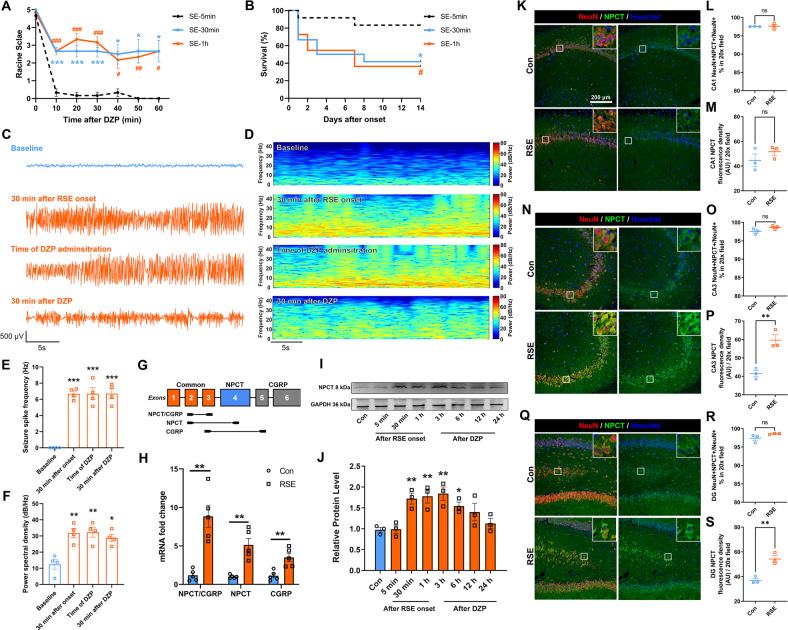
Hippocampal NPCT is overexpressed in CA3 pyramidal neurons in rat model of RSE. **A** Racine scale after DZP administration in SE lasting different durations. Repeated measure two-way ANOVA with Tukey’s post hoc analyses. *n* = 6 rats in each group. *P < 0.05, ***P < 0.001, SE-30 min vs. SE-5 min; ^#^*P* < 0.05, ^# #^*P* < 0.01, ^# # #^*P* < 0.001, SE-1h vs. SE-5 min. **B** Survival curves of animals with SE lasting different durations. Log-rank test. *n* = 12 rats in each group. *P < 0.05, SE-30 min *vs*. SE-5 min; ^#^*P* < 0.05, SE-1h *vs*. SE-5 min. **C** Representative hippocampal EEG traces and (**D**) corresponding EEG power spectrum within epoch length of 30 s at baseline before pilocarpine, 30 min after RSE onset, time when DZP was administered, and 30 min after DZP administration. **E** EEG seizure spike frequency and (**F**) power spectral density at different time points in the rat RSE model. One-way ANOVA with Turkey’s post hoc analyses. *n* = 4 rats. **G** Schematic of the *Calca* gene showing exons 1–3 shared by NPCT and CGRP, NPCT-specific exon 4, and CGRP-specific exons 5,6. Locations of primer sets are shown to measure NPCT + CGRP (exon 2 and 3), exclusive NPCT (exon 2 and 4), and exclusive CGRP (exon 3 and 5). Exon 4 is removed from the mRNA of CGRP. **H** The mRNA fold change of total NPCT/CGRP, exclusive NPCT, and exclusive CGRP after 1 h of RSE in rat hippocampus. Student’s *t*-test (with Welch’s correction for NPCT/CGRP and exclusive NPCT). *n* = 5 rats in each group. **I**, **J** Hippocampal NPCT protein levels at different time points in the RSE model. One-way ANOVA with Dunnett’s post hoc analyses. *n* = 3 rats in each group. **K** Representative images showing the expression of NPCT in CA1 neurons. Both the percentage of NPCT-containing neuron to total neuron number (**L**) and the NPCT fluorescence density within CA1 neurons (**M**) were calculated. Student’s *t*-test. *n* = 3 rats in each group. **N** Representative images showing the expression of NPCT in CA3 neurons. Both the percentage of NPCT-containing neuron to total neuron number (**O**) and the NPCT fluorescence density within CA3 neurons (**P**) were calculated. Student’s *t*-test. *n* = 3 rats in each group. **Q** Representative images showing the expression of NPCT in DG neurons. Both the percentage of NPCT-containing neuron to total neuron number (**R**) and the NPCT fluorescence density within DG neurons (**S**) were calculated. Student’s *t*-test. *n* = 3 rats in each group. *P < 0.05, ** *P* < 0.01, ***P < 0.001, compared with control group in the above figures excluding (**A**, **B**). All data are presented as mean ± SEM. AU arbitrary unit, CA1 field CA1 of the hippocampus, CA3 field CA3 of the hippocampus, DG dentate gyrus, DZP diazepam, RSE refractory status epilepticus.

Refractoriness of seizure increases as seizure prolongs [5]. To establish the RSE model, we determined the relationship of diazepam (DZP) resistance with seizure duration in the lithium-pilocarpine model. DZP was ineffective to halt seizure behavior in SE persisting 30 min or 1 h rather than 5 min (Fig. 2A). Moreover, the mortalities of SE lasting 30 min and 1 h were similar and significantly higher than that of SE lasting 5 min (Fig. 2B), consistent with clinical observations that RSE caused significantly higher mortality [3]. Drug resistance was further confirmed by the hippocampal electroencephalogram (EEG) in SE lasting 1 h. Both seizure spike frequency and total spectral power significantly increased after the onset of SE and kept higher than baseline until 30 min after DZP (Fig. 2C–F), meeting the definition of drug resistance [15]. Thus, in the following experiments, we chose seizure lasting 1 h as the RSE model.

We next explored the hippocampal NPCT expression in this RSE model. Considering the *Calca* gene encoding NPCT has two transcripts (NPCT and calcitonin gene-related peptide, CGRP) due to alternative splicing, we designed primer sets to specifically detect the total mRNA of NPCT and CGRP, exclusive NPCT, and exclusive CGRP, according to the arrangement of exons (Fig. 1G) [16]. RSE triggered significant transcription of the exclusive mRNA of NPCT by around 5-fold (Fig. 2H). Moreover, the hippocampal NPCT protein levels started to rise 30 min after RSE onset and kept overexpressed until 6 h after DZP (Fig. 2I, J and supplementary file of uncropped western blots). To further confirm the cellular origin of the overexpressed NPCT, NPCT was co-stained with markers of neurons, astrocytes, and microglia. We found that RSE induced a significant increase of the NPCT fluorescence density in neurons of CA3 and DG (Fig. 2K–S) without altering the proportion of neurons expressing NPCT (Fig. 2L, O, R). Further morphological analysis confirmed that in CA3 NPCT was upregulated in the pyramidal neurons (Fig. 2N, insets), and in DG NPCT was increased in pyramidal neurons protruded from CA3 (Fig. 2Q, insets). RSE did not alter the expression of NPCT in either astrocyte or microglia (Figure S5). Taken together, these results demonstrated that RSE triggered specific overexpression of NPCT in hippocampal CA3 pyramidal neurons.

### NPCT promotes OXPHOS and facilitates ATP production in primary hippocampal neurons

**Fig. 3:**
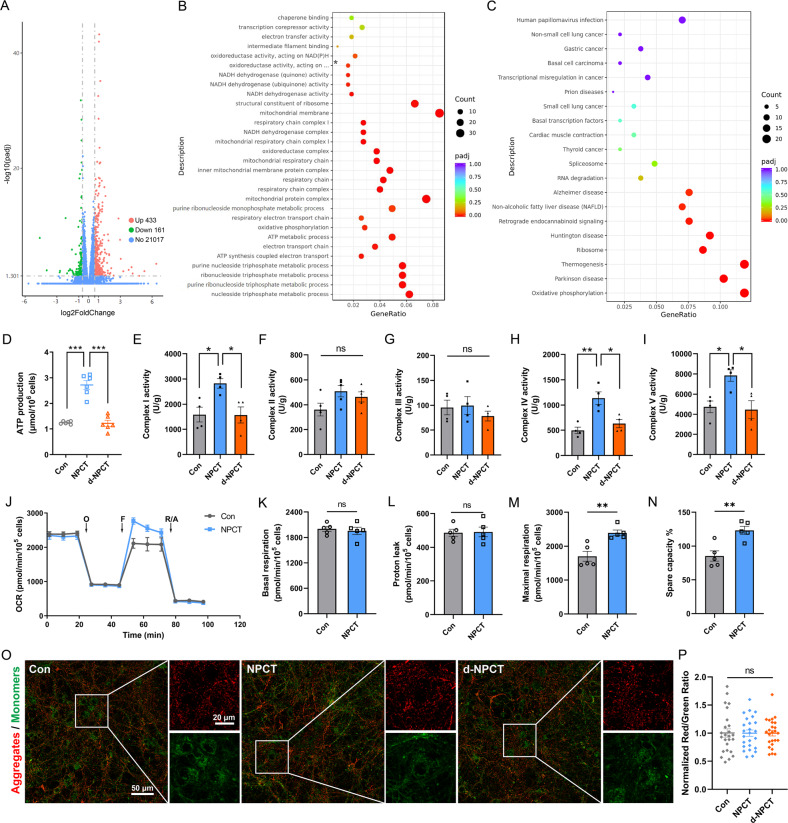
NPCT facilitates OXPHOS and ATP production in primary hippocampal neurons. **A** Volcano plots showing DEGs in NPCT-stimulated primary hippocampal neurons. **B** GO enrichment analysis of DEGs. Asterisk represents “oxidoreductase activity, acting on NAD(P)H, quinone or similar compound as acceptor”. **C** KEGG enrichment analysis of DEGs. **D** The influence of NPCT on intracellular ATP levels in primary hippocampal neurons. Welch’s ANOVA with Dunnett’s T3 post-hoc analyses. Representative result from 3 independent experiments. ***P < 0.001. **E**–**I** The influences of NPCT on the activities of mitochondrial respiratory chain complexes I-V in primary hippocampal neurons. One-way ANOVA with Turkey’s post hoc analyses. *n* = 4 biological replicates. *P < 0.05, **P < 0.01. **J** Representative OCR of primary hippocampal neurons exposed to 1 nM NPCT for 24 h, determined by the mitochondrial stress test. OCR was measured before and after sequential injections of oligomycin (O), FCCP (F), and rotenone with antimycin A (R/A). *n* = 3 independent experiments. **K**–**N** Basal respiration, proton leak, maximal respiration, and spare capacity% were calculated from (**J**) as described in the Methods section. Student’s *t*-test. **P < 0.01. **O**, **P** JC-1 staining showing the mitochondrial membrane potential of primary hippocampal neurons stimulated with 1 nM NPCT for 24 h. One-way ANOVA with Turkey’s post hoc analyses. *n* = 25 fields from 3 biological replicates. All data are presented as mean ± SEM. DEGs differentially expressed genes, OCR oxygen consumption rate, OXPHOS oxidative phosphorylation.

To explore the functions of NPCT to neurons, high-throughput RNA-sequence was performed in primary hippocampal neurons stimulated with NPCT. A total of 594 genes were differentially expressed with 433 upregulated and 161 downregulated (Fig. 3A). GO and KEGG enrichment analyses were performed to differentially expressed genes (DEGs). In GO analysis, the top significant enrichments in biological process, cellular component, and molecular function were “nucleoside triphosphate metabolic process”, “mitochondrial protein complex”, and “structural constituent of ribosome”, respectively (Fig. 3B). While in KEGG analysis, the most enriched term was “oxidative phosphorylation” (Fig. 3C). Thus, we deduced that NPCT had significant impacts on mitochondrial respiratory chain complexes and OXPHOS.

To verify and confirm the exact influences of NPCT on neuronal OXPHOS, we measured the intracellular ATP level and the activities of mitochondrial respiratory chain complexes I-V after NPCT stimulation. NPCT significantly increased the intracellular ATP level by approximately 2-fold (Fig. 3D) and upregulated the activities of mitochondrial respiratory chain complexes I, IV, and V in primary hippocampal neurons (Fig. 3E–I). Heat-deactivated NPCT (d-NPCT) did not have any of these effects (Fig. 3D–I), excluding the possibility of unspecific exogeneous stimulating effects. Oxygen consumption rate (OCR) was further measured using the Seahorse XFe24 (Fig. 3J). We found that NPCT significantly increased neuronal maximal respiration and spare respiration capacity% (Fig. 3K–N). Mitochondrial membrane potential (MMP) of primary hippocampal neurons was also investigated and we found that MMP was unaltered after NPCT treatment (Fig. 3O, P). Taken together, these results uncovered that NPCT facilitated OXPHOS and ATP production, suggesting the essential role of NPCT in neuronal survival.

Meanwhile, we also performed RNA-sequence to primary hippocampal neurons stimulated with PCT, the parent peptide of NPCT. To our surprise, only a minority of DEGs was shared between neurons stimulated with NPCT and PCT (Supplementary Fig. S6A, B). GO analysis revealed that DEGs induced by PCT were mainly enriched in “immune effector process”, “extracellular matrix”, and “glutathione transferase activity” in categories of biological process, cellular component, and molecular function, respectively (Supplementary Fig. S6C). In KEGG analysis, the top two enriched terms include “staphylococcus aureus infection” and “cytokine-cytokine receptor interaction” (Supplementary Fig. S6D). These results confirmed the established inflammatory roles of peripheral PCT in CNS neurons, and also revealed the contrasting differences in the functions between PCT and NPCT in neurons.

### NPCT has multiple neurotrophic effects on primary hippocampal neurons

**Fig. 4:**
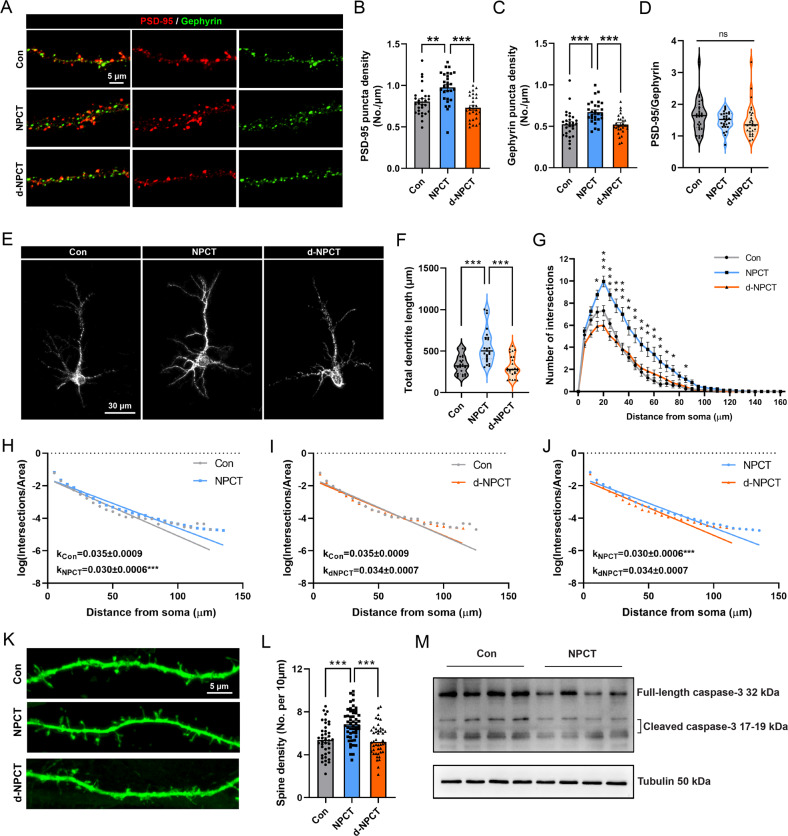
NPCT has multiple neurotrophic effects on primary hippocampal neurons. **A** Representative images showing the influences of incubation with 1 nM NPCT and d-NPCT for 24 h on the excitatory synaptic marker PSD-95 and inhibitory synaptic marker gephyrin in primary hippocampal neurons. **B**–**D** Quantifications of the density of PSD-95 puncta, gephyrin puncta, and the ratio of PSD-95/gephyrin. One-way ANOVA with Turkey’s post hoc analyses in (**B**, **C**) and Kruskal–Wallis *H* test in (**D**). *n* = 28 dendrites from 3 independent experiments in each group. **P < 0.01, ***P < 0.001. **E** Representative images demonstrating the neuritogenesis effects of 1 nM NPCT and d-NPCT incubation for 24 h in primary hippocampal neurons. **F** Statistical analysis showing the effects of NPCT on the total dendrite length. Kruskal–Wallis *H* test with Dunn’s post hoc analyses. *n* = 25 neurons from 3 independent experiments in each group. ***P < 0.001. **G** Linear sholl analysis showing that NPCT facilitated dendritic arborization in primary hippocampal neurons. Repeated-measure two-way ANOVA with Dunnett’s post hoc analyses. *n* = 25 neurons from 3 independent experiments in each group. *P < 0.05, **P < 0.01, ***P < 0.001, compared with control group. **H–J** Semi-log sholl analyses demonstrating that NPCT increased the complexity of dendritic arborization. A fitting line was generated by simple linear regression for each group and the k coefficient, calculated by multiplying negative one with the slope of the fitting line, was compared between groups. *n* = 25 neurons from 3 independent experiments in each group. ***P < 0.001, compared between groups. **K** Representative images showing the spinogenesis effects of NPCT in primary hippocampal neurons. **L** Statistical analysis demonstrating that NPCT increased the spine density. One-way ANOVA with Turkey’s post hoc analyses. *n* = 40 dendrites, 52 dendrites, and 44 dendrites from 3 independent experiments in the control, NPCT, and d-NPCT group, respectively. ***P < 0.001. **M** Immunoblots showing the effects of NPCT on the expression of full-length and cleaved caspase-3. *n* = 4 independent experiments. All data except (**D**, **F**) are presented as mean ± SEM. Data in (**D**, **F**) are presented as median with IQR.

The imbalance of excitatory and inhibitory synapses underlies the neuronal death after RSE [17, 18]. Next, we investigated the effects of NPCT on PSD-95, the excitatory synapse marker, and gephyrin, the inhibitory synapse marker, in primary hippocampal neurons. We found that NPCT simultaneously increased the density of PSD-95 and gephyrin (Fig. 4A–C) without altering the ratio of PSD-95/gephyrin (Fig. 4D), suggesting that NPCT promoted excitatory and inhibitory synapse formation without altering excitatory/inhibitory (E/I) balance, and raised the possibility that NPCT might promote neuritogenesis and spinogenesis, which are the structural bases for synapse alterations. As expected, NPCT significantly increased the total dendrite length (Fig. 4E, F) and facilitated dendritic arborization revealed by both linear (Fig. 4G) and semi-log (Fig. 4H–J) sholl analyses. NPCT also increased spine density (Fig. 4K, L). Lastly, we investigated the expression of caspase-3, the apoptotic effector, after NPCT stimulation. We found that NPCT decreased both the full-length caspase-3 and cleaved caspase-3 (Fig. 4M and supplementary file of uncropped western blots). Taken together, these results unraveled that NPCT had multiple neurotrophic effects on hippocampal neuronal survival without disturbing E/I balance, including enhancing synaptogenesis, neuritogenesis, and spinogenesis, and suppressing apoptotic effector.

### Immunoneutralization of NPCT causes more neuronal death in 0-Mg^2+^ in vitro SE model

**Fig. 5:**
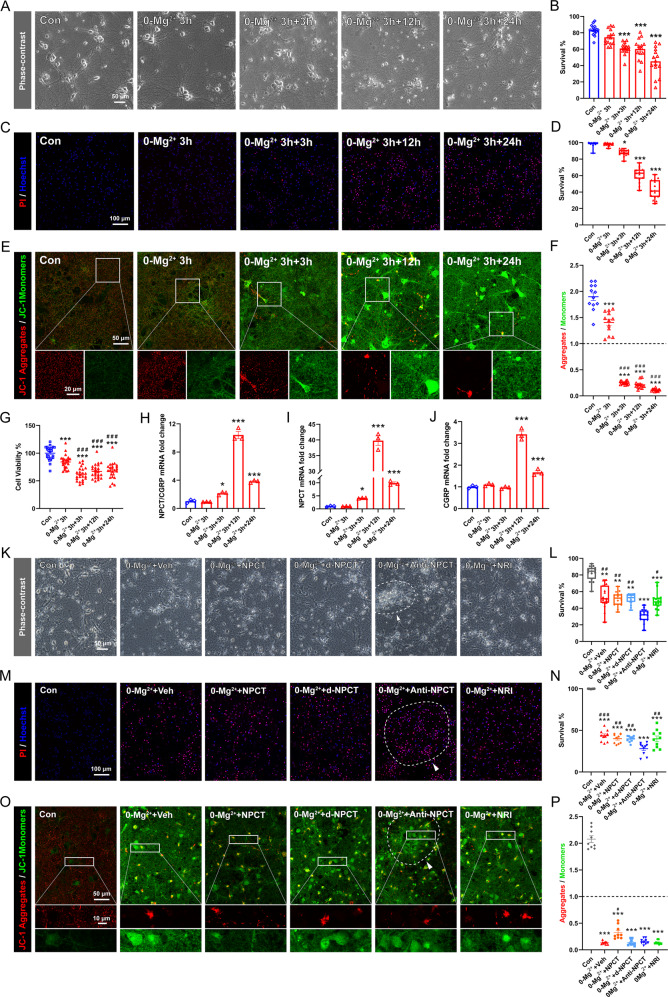
Immunoneutralization of NPCT in the in vitro SE model increased neuronal death. **A**, **B** Morphological analysis of neuronal death based on phase-contrast images captured at the end of 3 h 0-Mg^2+^ exposure (0-Mg^2+^ 3 h), 3 h after the end of 0-Mg^2+^ exposure (0-Mg^2+^ 3 h + 3 h), 12 h after the end of 0-Mg^2+^ exposure (0-Mg^2+^ 3 h + 12 h), and 24 h after the end of 0-Mg^2+^ exposure (0-Mg^2+^ 3 h + 24 h). Welch’s ANOVA with Dunnett’s T3 post-hoc analyses. *n* = 15 fields from 3 biological replicates. ***P < 0.001, compared with control group. **C**, **D** PI/Hoechst staining to investigate neuronal death at different time points as those in (**A**, **B**). Kruskal–Wallis *H* test with Dunn’s post hoc analyses. *n* = 15 fields from 3 biological replicates. *P < 0.05, ***P < 0.001, compared with control group. **E**, **F** JC-1 staining to detect MMP at different time points as those in (**A**, **B**). Welch’s ANOVA with Dunnett’s T3 post-hoc analyses. *n* = 12 fields from 3 biological replicates. ***P < 0.001, compared with control group; ^###^*P* < 0.001, compared with 0-Mg^2+^ 3 h group. **G** CCK-8 assays investigating neuronal viability at different time points as those in (**A**, **B**). One-way ANOVA with Turkey’s post hoc analyses. *n* = 24 wells from 3 independent experiments. ***P < 0.001, compared with control group; ^###^*P* < 0.001, compared with 0-Mg^2+^ 3 h group. **H**–**J** Relative mRNA fold change of the total mRNA of NPCT/CGRP, the exclusive NPCT mRNA, and the exclusive CGRP mRNA. One-way ANOVA with Dunnett’s post hoc analyses. *n* = 3 independent experiments. *P < 0.05, ***P < 0.001, compared with control group. **K**, **L** Morphological analysis of neuronal death based on phase-contrast images captured at 24 h after 3 h 0-Mg^2+^ exposure. Kruskal–Wallis *H* test with Dunn’s post hoc analyses. *n* = 15 fields from 3 biological replicates. ** *P* < 0.01, ***P < 0.001, compared with control group; ^#^*P* < 0.05, ^##^*P* < 0.01, compared with 0-Mg^2+^+Anti-NPCT group. **M**, **N** PI/Hoechst staining to investigate neuronal death after drug administration. Welch’s ANOVA with Dunnett’s T3 post-hoc analyses. *n* = 10 fields from 3 biological replicates. ***P < 0.001, compared with control group; ^##^*P* < 0.01, ^###^*P* < 0.001, compared with 0-Mg^2+^+Anti-NPCT group. **O**, **P** JC-1 staining to detect MMP after drug administration. Welch’s ANOVA with Dunnett’s T3 post-hoc analyses. *n* = 9 fields from 3 biological replicates. ***P < 0.001, compared with control group; ^#^*P* < 0.05, compared with any other group except the control group. Dotted line and arrowheads in (**K**, **M**, **O**) denote detached neurons mixed with piled up debris. All data except (**D**, **L**) are presented as mean ± SEM. Data in (**D**, **L**) are presented as median with IQR. MMP mitochondrial membrane potential, NRI nonimmune rabbit IgG.

To examine the roles of NPCT in neuronal death after seizure, we induced in vitro SE lasting 3 h in primary hippocampal neuronal network with 0-Mg^2+^ buffer as previously described [19] (please refer to materials and methods for details). The successful establishment of this in vitro SE model was confirmed by repetitive [Ca^2+^]_i_ oscillations as revealed by calcium imaging (Supplementary Fig. S7). Both phase-contrast morphological analysis (Fig. 5A, B) and PI staining (Fig. 5C, D) demonstrated significant neuronal death after in vitro SE. However, survival immediately at the end of SE remained unaltered (Fig. 5A–D). MMP was monitored and we found that MMP decreased immediately after in vitro SE until 24 h later (Fig. 5E, F), which was accompanied by simultaneous decrease in cellular viability monitored with CCK8 (Fig. 5G). It was noticeable that MMP and cellular viability decreased earlier than the death of neurons, suggesting that bioenergy failure occurred prior to final neuronal death. We next investigated the transcription of *Calca* gene in the in vitro SE model. The mRNA of total NPCT/CGRP and the exclusive NPCT mRNA significantly increased from 3 h, peaked at 12 h, and remained upregulated until 24 h after in vitro SE (Fig. 5H, I). By contrast, the mRNA of CGRP remained unaltered within the first 3 h after in vitro SE (Fig. 5J). Importantly, the exclusive NPCT mRNA peaked by 39.78 folds of the baseline, while the exclusive mRNA of CGRP only peaked by 3.42 folds and the total mRNA 10.42 folds (Fig. 5H–J), suggesting that neuronal death after in vitro SE triggered a shift of the alternative splicing of *Calca* gene towards more NPCT production. We hypothesized that this upregulation of NPCT was an emergent self-rescuing response during the dying process.

To test this hypothesis, NPCT immunoneutralization antibody (anti-NPCT) was added immediately after the end of SE to antagonize NPCT. NPCT peptide was also added to see whether exogenous NPCT supplementation was helpful to halt neuronal death. We found that immunoneutralization of NPCT significantly exacerbated neuronal conditions and increased neuronal death (Fig. 5K–N), with neurons treated with anti-NPCT detaching from the surface and the debris of neurons piled together (Fig. 5K, M, O; dotted-line circles pointed by arrowheads). However, exogenous NPCT did not increase the survival (Fig. 5K–N). The effects of anti-NPCT and exogenous NPCT on MMP were also monitored. Anti-NPCT did not alter the MMP while exogenous NPCT supplementation preserved MMP at 24 h after in vitro SE (Fig. 5O, P). Taken together, these results suggested that the overexpressed NPCT acted as a neuroprotectant after in vitro SE. The ineffectiveness of exogenous NPCT to inhibit neuronal death could be due to the ceiling effects of endogenously overexpressed NPCT.

### Immunoneutralization of NPCT in rat RSE model exacerbated hippocampal neuronal death

**Fig. 6:**
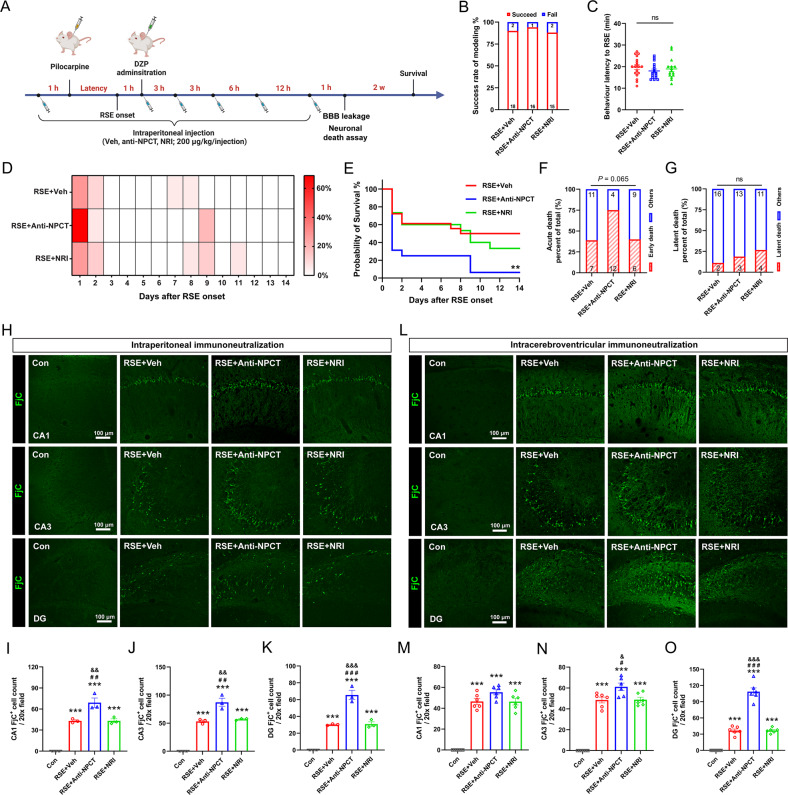
Immunoneutralization of NPCT increased hippocampal neuronal death in rat RSE model. **A** Schematic showing the procedure of peripheral NPCT immunoneutralization. **B** Success rate of modeling in the three groups. **C** Behavioral latency to RSE onset in animals receiving intraperitoneal anti-NPCT. One-way ANOVA test. *n* = 15–18 rats in each group. **D** Heatmap showing the percentages of daily death of animals receiving intraperitoneal anti-NPCT within 14 d after RSE onset. **E** Survival curves comparing the mortality within 14 d after RSE onset. Log-rank test. *n* = 15–18 rats in each group. **P < 0.01, compared with RSE + Veh group. **F**, **G** The proportion of rats with acute death and latent death respectively. Chi-square test. *n* = 15–18 rats in each group. **H**–**K** FjC staining showing degenerating neurons in CA1, CA3, and DG after intraperitoneal anti-NPCT administration in RSE. One-way ANOVA with Turkey’s post hoc analyses. *n* = 3 rats in each group. ***P < 0.001, compared with control group; ^# #^*P* < 0.01, ^###^*P* < 0.001, compared with RSE + Veh group; ^&&^*P* < 0.01, ^&&&^*P* < 0.001, compared with RSE + NRI group. **L**–**O** FjC staining showing degenerating neurons in CA1, CA3, and DG after intracerebroventricular anti-NPCT administration in RSE. One-way ANOVA with Tukey’s post hoc analyses. *n* = 6 rats in each group. ***P < 0.001, compared with control group; ^#^*P* < 0.05, ^###^*P* < 0.001, compared with RSE + Veh group; ^&^*P* < 0.05, ^&&&^*P* < 0.001, compared with RSE + NRI group. All data except (**B**, **F**, **G**) are presented as mean ± SEM. Data in (**B**, **F**, **G**) are presented as percentages with the numbers in columns representing the animal number. NRI nonimmune rabbit IgG.

Next, we investigated the necessity of NPCT to inhibit neuronal death in rat RSE model. Considering the disruption of blood-brain barrier (BBB) induced by RSE provides entrance for peripheral IgG to infiltrate into the brain parenchyma [20], intraperitoneal (i.p.) intermittent bolus injections of anti-NPCT were conducted as Fig. 6A, with the total dose consistent with that previously used in rat sepsis model [21]. RSE induced significant extravasation of IgG in hippocampus and anti-NPCT did not affect the extent of extravasation (Supplementary Fig. S8). Peripheral immunoneutralization of NPCT did not influence the success rate of modeling or behavioral latency to RSE onset (Fig. 6B, C). A bimodal distribution of death after RSE was noticed in each group (Fig. 6D), with the first peak within 48 h (acute death) and the second peak at 1 week after RSE (latent death). Log-rank test showed that the survival overtime was significantly lower in rats receiving anti-NPCT (Fig. 6E). Further analysis demonstrated that anti-NPCT remarkably increased acute death without affecting latent death (Fig. 6F, G). Taken together, these data demonstrated that NPCT is endogenous protectant against death after RSE.

We next investigated the neuronal death in hippocampus. Consistent with in vitro SE model, we found that i.p. anti-NPCT induced significantly more FjC^+^ neurons in both the CA1, CA3, and DG compared to RSE animals receiving vehicle (Fig. 6H–K). To antagonize central NPCT more specifically, anti-NPCT was further administered intracerebroventricularly. Compared with RSE animals receiving vehicle, i.c.v. immunoneutralization of NPCT significantly increased the number of FjC^+^ neurons in CA3 and DG, sparing those in CA1 (Fig. 6L–O). Further morphological inspection demonstrated that the increased FjC^+^ neurons in DG were pyramidal neurons protruded from CA3, overlapping with the cellular types overexpressing NPCT during RSE (Fig. 2N, Q). Taken together, these results demonstrated that antagonizing NPCT caused significant neuronal death, suggesting that NPCT protects against hippocampal neuronal death after RSE.

### Intracerebroventricular immunoneutralization of NPCT caused serious hippocampal EEG exhaustion and ATP depletion in RSE

**Fig. 7:**
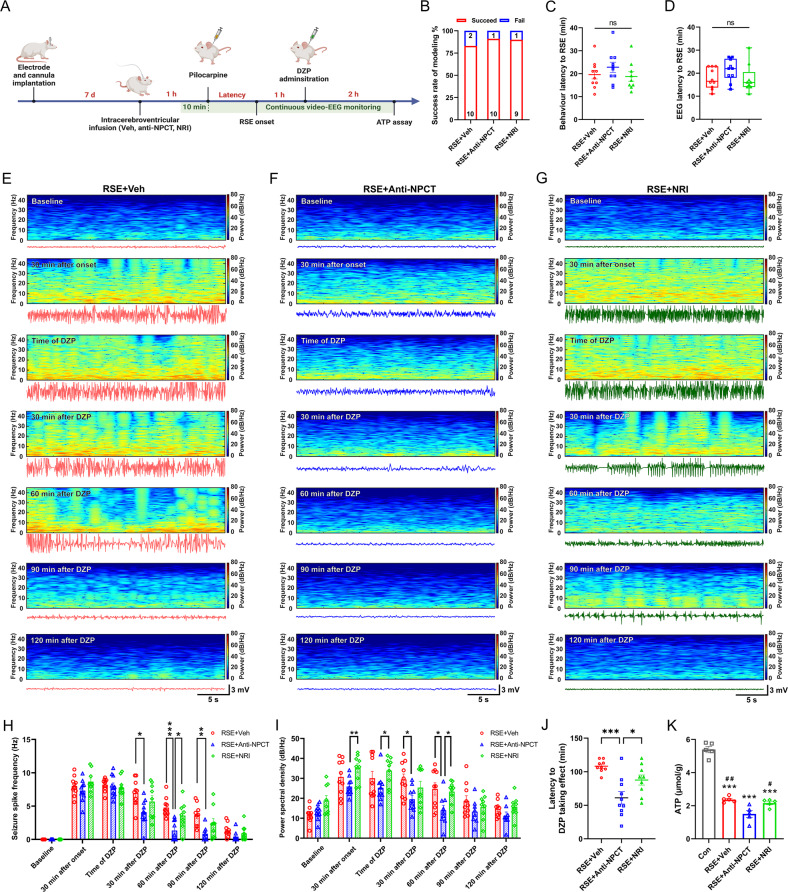
Intracerebroventricular (i.c.v.) immunoneutralization of NPCT caused serious EEG power exhaustion and hippocampal ATP depletion in RSE. **A** Schematic showing the procedure of i.c.v. NPCT immunoneutralization. **B** The success rate of modeling was similar among the three groups. Chi-square test. *n* = 10–12 rats in each group. **C**, **D** The behavior and EEG latencies to RSE onset were similar among the three groups. One-way ANOVA test in (**C**) and Kruskal–Wallis *H* test in (**D**). *n* = 9–10 rats in each group. **E**–**G** Representative hippocampal EEG power spectrums and corresponding EEG tracs in RSE animals receiving vehicle, anti-NPCT, and nonimmune rabbit IgG (NRI) in epochs of 30 s at different time points, i.e., baseline before pilocarpine, 30 min after onset, time of DZP administration, 30 min after DZP administration, 90 min after DZP administration, and 120 min after DZP administration. **H** The influences of i.c.v. immunoneutralization of NPCT on the seizure spike frequency and EEG spectral power (**I**) at different time points as those in (**E**). Mixed effects model with Turkey’s post hoc analyses. *n* = 9–10 rats in each group. *P < 0.05, **P < 0.01, ***P < 0.001, compared as graphed. **J** The latency to DZP taking effects in each group. One-way ANOVA with Turkey’s post hoc analyses. *n* = 9–10 rats in each group. *P < 0.05, ***P < 0.001. **K** Hippocampal ATP levels at the end of video-EEG monitoring. One-way ANOVA with Turkey’s post hoc analyses. *n* = 5 rats in each group. ***P < 0.001, compared with control group; ^#^*P* < 0.05, ^##^*P* < 0.01, compared with RSE + Anti-NPCT group. All data except (**B**, **D**) are presented as mean ± SEM. Data in (**B**) are presented as percentages with the numbers in columns representing the animal number. Data in (**D**) are presented as median with IQR.

To further validate the involvement of NPCT-mediated OXPHOS and energy production in its neuroprotective effects in RSE, anti-NPCT was intracerebroventricularly administered and continuous behavior-EEG and hippocampal ATP were monitored as Fig. 7A. I.c.v. immunoneutralization of NPCT did not influence the success rate of modeling, or the behavioral and EEG latency to RSE onset (Fig. 7B–D). Noticeably, one RSE animal receiving vehicle died after a sequence of violent generalized tonic-clonic seizures at 60 min after DZP (Supplementary Fig. S9A). Two animals in the RSE + Anti-NPCT group died at 54 min and 75 min after DZP (Supplementary Fig. S9B) before which their EEG had already exhausted electrophysiologically. Thus, their EEG data after death were not included in further analysis.

I.c.v. immunoneutralization of NPCT did not influence the EEG seizure spike frequency before DZP treatment but significantly reduced spike frequency from 30 min to 90 min after DZP (Fig. 7E–H). In EEG spectral power analysis, i.c.v. immunoneutralization of NPCT not only decreased the total EEG power from 30 min to 60 min after DZP but also lowered the EEG power during RSE (Fig. 7E–G, I). The latency to DZP taking effect was significantly shorter in animals receiving anti-NPCT (Fig. 7J). Collectively, these results demonstrated that immunoneutralization of NPCT induced serious hippocampal EEG power exhaustion and faster seizure wane in RSE animals, suggesting insufficient energy supply to sustain seizure activity. We next detected the intrahippocampal ATP levels at the end of behavior-EEG monitoring. We found that i.c.v. immunoneutralization of NPCT further exacerbated the hippocampal ATP depletion compared with RSE animals receiving vehicle (Fig. 7K). Taken together, these results suggested that immunoneutralization of NPCT caused significant hippocampal ATP depletion and serious EEG power exhaustion, which underlay the exacerbated neuronal death after RSE.

## Discussion

The current study advanced our knowledge about the distribution of NPCT in CNS to a great deal. In their ground-breaking work, Ojeda et al. detected PCT-like immunoreactivity in the rat hypothalamus [22]. Tavares et al. further discovered the existence of NPCT in the rat paraventricular nucleus (PVN) [10] and arcuate nucleus [12] as well as in mice cortex and hippocampus [23]. In the current study, we demonstrated that NPCT was extensively distributed throughout the gray matters of rat brain. Apart from the hypothalamus, high levels of NPCT were also found in the orbital cortices, median thalamic nuclei, hippocampus, olfactory cortices, and midbrain oculomotor nucleus. This extremely extensive distribution of NPCT implies its fundamental and shared functions for neurons irrespective of locations. Following findings that NPCT regulated neuronal OXPHOS and exerted neurotrophic effects accord with this implication. Apart from neurons, NPCT was also detected in astrocytes. Moreover, previous studies reported the secretion of NPCT from thyroid C cells [24]. The glial and peripheral distribution of NPCT is not an eccentric, but a common phenomenon for many neuropeptides [25]. Taken together, the current study provided a reference atlas of CNS NPCT distribution for future investigation.

The brain is a highly energy-demanding organ [26]. A characteristic of brain energy is the tight coupling between neural activity with local blood flow, also known as neurovascular coupling [27]. We found that NPCT, as a neuropeptide, served as a regulator of the brain energy status by directly controlling neuronal OXPHOS and ATP production. Neuropeptides as energy modulator have their unique features. Unlike classic neurotransmitters released acutely upon stimulation and deactivated quickly post actions, neuropeptides could be released constitutively, diffuse in the form of volume transmission, and are terminated through enzymatic degradation [25]. This means that neuropeptides have longer effective time and working distance. In the case of energy modulator, these features represent a chronic and holistic regulation, unlike the immediate and precise coupling between neuronal activity and blood flow.

Sustaining seizure activity represents a metabolic burden pushing brain energy supply to its limits [28]. In our study, the overexpression of NPCT triggered by RSE represented an emergent response to the high energy demand during sustaining seizure. Previous studies reported that i.c.v. NPCT administration induced catabolic effects via activating the hypothalamic-pituitary adrenal (HPA) axis, resulting in increased plasma corticosterone [10, 11], subsequently preserving plasma glucose for the brain to counter stress [29]. Combined with current findings, we summarized that during RSE, NPCT served as an energy booster acting both centrally and peripherally, either by directly promoting neuronal OXPHOS and ATP production in situ in the brain, or by activating HPA axis to mobilize the whole body to transport energy in the form of glucose to the brain.

Severe neuronal death is the most prominent pathological consequence of RSE [30]. Mitochondrial dysfunction and ATP depletion is the convergences of factors contributing to neuronal death [8]. Energy failure jeopardized neuronal ability to maintain ionic homeostasis, consequently leading to depolarization, hyperexcitability and neuronal death [8]. Previous studies reported that increasing ATP production during seizure activity by providing OXPHOS substrate prevented neuronal death [31, 32]. Complementary to these results, we found that exacerbated ATP depletion induced by anti-NPCT was associated with more neuronal death, confirming the central pathological role of energy failure in neuronal death after RSE. Besides neuronal death, energy failure and ATP depletion after RSE also contributes to epileptogenesis. On the one hand, insufficient ATP supply would affect Na^+^/K^+^ ATPases and increase neuronal excitability and seizure susceptibility via lowering membrane potential [33, 34]. On the other hand, ATP depletion would disrupt the mobilization of the synaptic vesicle reserve pool of parvalbumin-positive interneurons, the most abundant cortical GABAergic inhibitory interneurons, leading to insufficient GABA release and hyperexcitability of neural circuits, and thus contribute to epileptogenesis [6, 35–37]. In our study, although exogenous NPCT supplementation could not reverse neuronal death after RSE, it was still protective for the mitochondrial function in the surviving neurons, raising the possibility that NPCT supplementation might protect against epileptogenesis via preserving mitochondrial OXPHOS and ATP supply. Further studies are needed to test this therapeutic possibility.

In the current study, NPCT was overexpressed in the CA3 pyramidal neurons and i.c.v. immunoneutralization of NPCT caused significant death of these neurons. Why CA3 pyramidal neurons? In the intrinsic trisynaptic hippocampal circuits [38], CA3 pyramidal neurons receive signals from DG granule cells and transfer them to CA1 pyramidal neurons. Moreover, CA3 also sends collaterals onto other CA3 neurons [38], back-projections to DG granule cells [39], and commissural connections to the contralateral CA3 and CA1 [40]. These reciprocal connections make CA3 pyramidal neurons the relay hub and reinforcement of hippocampal circuitry activity in epilepsy [41], conferring “energy privilege” on these neurons during seizure activity. Thus, during RSE, NPCT was specifically upregulated in CA3 pyramidal neurons to meet their extra energy demand, and the energy depletion caused by anti-NPCT failed their need and starved them to die.

Another interesting finding in the study is that NPCT has multiple neurotrophic effects. Neurotrophins are a family of peptidergic regulators of neuronal survival, growth, and plasticity, with brain derived-neurotrophic factor (BDNF) the most characterized one [42]. BDNF facilitated dendritic outgrowth and spine formation [43], and also promoted neuronal survival and synaptogenesis [44]. In SE, BDNF could protect against cell damage [45]. The functions of NPCT were quite similar with those of BDNF, raising the possibility that NPCT might be a novel member of the neurotrophin family. Two possible mechanisms might underly the neurotrophic effects of NPCT. The first is that the neurotrophic effects of NPCT is the consequences of its facilitations to mitochondrial functions and energy production. Assembly of cytoskeleton for the development of synaptic compartments is energy-consuming and is supported by mitochondrial ATP production [46]. Sufficient amount and normal functioning of mitochondria are essential and supportive for the development and maintenance of dendrites, spines, and synapses [46, 47]. In the case of the NPCT, it promoted neuritogenesis, spinogenesis, and synaptogenesis via enhancing neuronal mitochondrial respiration and ATP production. The second mechanism of NPCT’s neurotrophic effects might be its suppression on caspase-3. Apart from its canonical function in mediating apoptosis, caspase-3 plays key roles in pruning dendrites and spines without causing cell death [48]. It was demonstrated that caspase deficiency suppressed the pruning of dendrites, leading to increased dendritic arborization [49]. Meanwhile, local activation of dendritic caspase-3 led to elimination of spines and retraction of dendrites without inducing cell death [50, 51]. More importantly, the BDNF pro-peptide, another neurotrophin family member, dramatically reduced dendritic spine density in hippocampal neurons in a caspase-3 dependent manner [52]. In our study, we found that NPCT significantly inhibited the expression of both the full-length and cleaved caspase-3 and increased dendritic arborization and spine density. This suggested that NPCT might exert its neurotrophic functions via suppressing the pruning effects of caspase-3. Future study is warranted to test these mechanisms and to further investigate the influences of NPCT on synaptic electrophysiological plasticity.

In the current study we provide first-hand evidences that NPCT might be functionally independent of its parent peptide PCT, clarifying previous claims that NPCT was the bioactive fragment responsible for the functions of PCT, which had not been experimentally verified directly [11, 13, 21]. Firstly, RNA-sequence results revealed that NPCT and PCT had contrasting effects on the transcriptome of primary hippocampal neurons, with DEGs stimulated with NPCT enriched in mitochondrial functions and DEGs stimulated with PCT enriched in inflammatory processes. This suggests that both NPCT and PCT are bioactive and their biological functions in neurons are distinct. Secondly, we found that NPCT decreased the expression of cleaved caspase-3 in primary hippocampal neurons and inhibited neuronal death after RSE, while PCT increased the level of cleaved caspase-3 and triggered neuronal death (data not shown), suggesting the opposite effects of the two peptides on neuronal survival. In the CNS, the biological activities of neuropeptide families are strictly regulated by proteinases to transform the parent peptides into products with similar, different, or terminated biological functions [25, 53, 54]. Thus, the parent peptide PCT and its products, including NPCT, calcitonin (CT, the classic calcium-modulating peptide cleaved from the middle part of PCT), and katacalcin (the fragment peptide cleaved from the carboxyl terminal of PCT) [55] comprised a neuropeptide family which has complex biological functions.

**Fig. 8:**
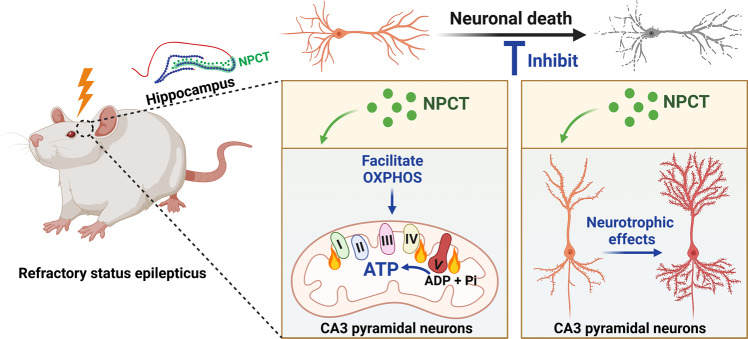
Schematic showing that RSE triggers overexpression of NPCT in hippocampal CA3 pyramidal neurons. NPCT protects hippocampal neuronal survival after RSE via facilitating mitochondrial OXPHOS and ATP production accompanied by multiple neurotrophic effects.

In conclusion, our study uncovered that NPCT was a neuropeptide distributed extensively in the brain. NPCT served as a potent modulator of neuronal OXPHOS and ATP production, and had multiple neurotrophic effects, promoting neuronal survival. During RSE, NPCT is overexpressed to facilitate neuronal ATP production and protect against neuronal death (Fig. 8). One major limitation of the study is that we didn’t identify the exact receptor of NPCT. Future studies are urgently needed to explore the neuronal receptor system of NPCT, as well as to translate the neuroprotectant effects of NPCT into the treatment of neurological diseases. Considering the full name of NPCT, aminoprocalcitonin, is too long and contains no connotation of biological functions, as well as its evolutionarily conserved amino acid sequence, extensive CNS distribution, fundamental roles in regulating energy homeostasis, and potential translational value for neurological diseases, we would like to propose a new nomenclature of this peptide, which is *Calenerin*. The prefix *Cal-* originates from the name of calcitonin, representing that it belongs to the calcitonin gene-encoding peptide family. The middle part *-ener-* originates from the Latin word *energia* that means energy in English. And the postfix *-in* represents its peptide property, which is a universal usage in the naming of neuropeptides.

## Materials and methods

### Animals and the establishment of SE model

Male Sprague-Dawley rats weighing 200–250 g were used in the study. Animals were housed in specific pathogen-free environment with 12-h light/ 12-h dark cycle and ad libitum access to water and rodent chow. All animal experiments were performed in accordance with the National Institutes of Health guidelines for the use of experimental animals and approved by the Animal Experiment Administration Committee of the Fourth Military Medical University, China.

Lithium-pilocarpine induced SE model was established as previously described [56]. Rats were injected with 3 meq/kg of lithium chloride (Sigma-Aldrich Cat# 62476, St Louis, MO, USA) intraperitoneally. SE was induced 18–20 h later by i.p. injection of pilocarpine (30 mg/kg; Cayman Chemical Cat# 14487, Ann Arbor, MI, USA). Methyl scopolamine (1 mg/kg; Aladdin Cat# S129958, Shanghai, China) was administered subcutaneously (s.c.) 30 min before pilocarpine to reduce the peripheral effects of pilocarpine. The severity of seizure was classified with Racine scale [57]: Grade 1, mouth and facial movements; Grade 2, head nodding; Grade 3, unilateral forelimb clonus; Grade 4, bilateral forelimbs clonus and rearing; Grade 5, generalized tonic-clonic seizure characterized by rearing and falling. The successful establishment of SE was defined as the first on-set seizure with at least grade 3 in Racine scale without returning to normal behavior. SE was allowed to continue for different durations (5 min, 30 min, 1 h) and was halted with i.p. administration of diazepam (DZP) (10 mg/kg; Jinyao pharmaceutical co., ltd. Cat# H12020957, Tianjin, China). Animals that didn’t develop SE were excluded from further analysis. Control animals received normal saline instead of pilocarpine, with other reagents administered similarly as SE animals. Randomization was not applicable in the study.

### Surgery and video-EEG monitoring

I.c.v. drug delivery and EEG monitoring were conducted via cannulas aimed at the lateral cerebral ventricle and electrodes aimed at the hippocampus, respectively. Briefly, rats were anesthetized with isoflurane (5% for induction and 2.5% for maintenance in oxygen at 1 L/min; RWD Cat# R510-22, Shenzhen, China) and were fixed on stereotaxic apparatus (RWD Cat# 68018, Shenzhen, China). A 24-gauge stainless cannula was stereotaxically implanted into the right lateral ventricle (AP −0.84 mm, ML 1.8 mm, DV 4.1 mm) and a 26-gauge obturator cap was inserted into the guide cannula. Monopolar insulated stainless-steel electrodes (Jingong Cat# QZY-2, Wenzhou, China) with diameter of 200 μm were stereotaxically implanted to the bilateral hippocampus (AP −5.6 mm, ML ± 4.5 mm, DV 4 mm). A reference electrode was placed within the cerebellum (AP −12 mm, ML 0 mm, DV 4 mm) as previously described [15]. The cannula and electrode assembly were secured on the skull with dental cement (SUN Medical co., ltd. Super-Bond C&B, Moriyama, Japan). After surgery, rats were given 5 ml warmed Ringer’s solution s.c., carefully placed on a heating pad to receive supplemental warmth and were monitored until they became conscious and ambulatory. Rats were housed individually and were allowed to recover for at least 7 d before seizure induction.

Simultaneous video and EEG recording was initiated 10 min before pilocarpine and was continued until 2 h after DZP treatment. Video of seizure behavior was recorded with a high-speed camara (Logitech C920 Pro, Nanjing, China), while EEG activity from the hippocampal electrodes was amplified, filtered (high pass: 0.1 Hz; low pass: 45 Hz), and recorded (sampling rate: 100 Hz) with an EEG monitoring system (SOLAR3000N, Beijing, China) as previously described [58]. SE onset was electrographically characterized by continuous spike/polyspike discharges with frequency > 3 Hz, while SE termination was defined as the time when EEG activity returned to baseline, and spiking activity became arrhythmic and < 2 Hz [59]. The DZP treatment was considered effective if there was electrographic termination of SE within 30 min after DZP i.p. injection, without electrographic recurrence within the next 90 min [15]. SE was considered as RSE if DZP was ineffective. EEG seizure spike frequency and the total EEG spectral power were calculated at the following time points in timeframe of 30 s: baseline before pilocarpine, 30 min after RSE onset, the time of DZP administration, 30 min after DZP, 60 min after DZP, 90 min after DZP, and 120 min after DZP. EEG data were analysed by an investigator blinded to the grouping of animals. The total power of EEG was calculated by the Welch’s method using the pwelch Matlab function within data epochs of 30 s [60]. EEG spectrograms were extracted using the Short-Time Fourier Transform (STFT) [61].

### NPCT peptide and NPCT immunoneutralization antibody preparation

Purified human NPCT (A^1^PFRSALESSPADPATLSEDEARLLLAALVQDY-VQMKASELEQEQEREGSSLDSPRS^57^; BACHEM Cat# 4030454, Bubendorf, Switzerland; BACHEM, RRID:SCR_013558) was used in the current study because the amino sequence is highly conserved between humans and rodents [62], and previous studies have used this NPCT in rats, which enabled us to compare our results with published works [11, 23, 63]. The rabbit polyclonal NPCT immunoneutralization antibody (anti-NPCT) was synthesized by GenScript, NJ, USA (RRID:SCR_002891). The antigen was a synthetic peptide corresponding to amino acids 69-82 (E^69^QEREGSSLDSPRS^82^) of human pre-procalcitonin (PPCT), the precursor peptide of human NPCT. This sequence corresponds to a specific and highly conserved carboxyl terminal segment of human NPCT (amino acids 44-57) and differs from that of the rat sequence (E^44^QEAEGSSLDSPRS^57^) by only one amino acid [62]. The antigen amino acid sequence does not have alignments with any other member of the CT/CGRP family. This immunoneutralization antibody had been verified to effectively block the activity of NPCT by previous studies [21, 23, 63].

NPCT and anti-NPCT were dissolved in phosphate buffer saline (PBS) or artificial cerebrospinal fluid (aCSF; 120 mM NaCl, 3.0 mM KCl, 1.2 mM CaCl_2_, 1.2 mM MgCl_2_, 0.67 mM NaH_2_PO_4_, and 0.3 mM Na_2_HPO_4_, pH 7.4) as stock solution, passed through 0.22 μm pore size filters (Millipore Cat# SLGPR33RB, MA, USA), aliquoted, and stored at −80 °C. As for the in vitro experiments using NPCT and anti-NPCT, fresh aliquots stocked in PBS were thawed, dissolved in maintenance medium to the tested concentrations (1 nM for NPCT [64] and 50 μg/ml for anti-NPCT [23]), and warmed to 37 °C before use. Heat-deactivated NPCT (1 nM, 90 °C for 1 h, d-NPCT) was used as negative control as previously described [21]. As for in vivo experiments using anti-NPCT, aliquots stocked in aCSF were dissolved in aCSF to the concentration of 0.4 μg/μl and warmed to 37 °C before use. Nonimmune rabbit IgG (NRI; Sigma-Aldrich Cat# I5006, RRID: AB_1163659, St Louis, MO, USA) of the same concentration was used as negative control as previously described [12, 23].

### I.c.v. injection

To investigate the roles of NPCT in hippocampal neuronal death after RSE, rats were intracerebroventricularly injected with anti-NPCT and NRI at 1 h before the administration of pilocarpine and 2 h after DZP administration, and brain samples were collected for Fluoro-Jade C (FjC) staining 24 h later. To investigate the roles of NPCT in hippocampal energy failure in RSE, anti-NPCT and NRI were intracerebroventricularly injected at 1 h before the administration of pilocarpine, followed by continuous video-EEG monitoring. Hippocampal samples were collected for ATP assays at the end of video-EEG monitoring. The cannula caps were removed and replaced with a 30-gauge stainless injection cannula connected to polyethylene tube attached to a 25 μl Hamilton microsyringe. 5 μl aCSF solutions containing 2 μg anti-NPCT or NRI were injected with a micro-injection pump (LONGER TJ-1A, Baoding, China) over 60 s and the injection cannula was held in place for another 60 s before slowly withdrawing. This amount of anti-NPCT was chosen because previous studies confirmed its capability to block the catabolic behavioral effects of central NPCT [12, 23].

### Primary hippocampal neuronal culture

Rat primary hippocampal neuronal cultures were prepared as previously described with slight modifications [65]. Foetuses were collected on embryonic day 17 and hippocampi were dissected and placed in ice-cold Ca^2+^ and Mg^2+^-free HEPES-buffered Hank’s salt solution (HBSS; Gibco Cat# 00468, CA, USA). Dissociated cells were seeded at a density of 40,000 cells/cm^2^ on plates or cover glasses that had been coated with poly-D-lysine (0.1 mg/ml; Sigma-Aldrich Cat# P6282, St Louis, MO, USA) and washed with H_2_O. Hippocampal neuronal cultures were maintained at 37 °C in a 5% CO_2_/95% air atmosphere and were grown in maintenance medium composed of neurobasal medium (NB; Gibco Cat# 21103-049, CA, USA) supplemented with 2% B27 (Gibco Cat# 17504-044, CA, USA), glutamine (2 mM; Gibco Cat# 25030-081, CA, USA), and penicillin-streptomycin (100 U/ml; Gibco Cat# 15140-122, CA, USA). Maintenance medium was changed in half volume every other day. Cells were cultured without mitotic inhibitors for 5 days in vitro (DIV) to investigate neurite outgrowth, 7 DIV for RNA-sequence, Seahorse XFe analyser, and mitochondria function assays, 14 DIV for in vitro model of SE, and 21 DIV for spinogenesis and PSD-95/gephyrin expression.

### In vitro model of SE

In vitro model of SE was induced in primary hippocampal neuronal cultures at 14 DIV as previously described [19]. Maintenance medium was replaced with physiological basal recording solution (pBRS) with or without MgCl_2_ (in mM): 145 NaCl, 2.5 KCl, 10 HEPES, 2 CaCl_2_, 10 glucose, and 0.002 glycine, pH 7.3, and osmolarity adjusted to 325 ± 5 mOsm with sucrose. In vitro SE was induced at 37 °C with 5% CO_2_/95% air, by exposing neurons to pBRS without MgCl_2_ (0-Mg^2+^ buffer) for 3 h, while control cultures were exposed to pBRS with 1 mM MgCl_2_ (control buffer) for the same period, after which the maintenance medium was changed back. pBRS with or without MgCl_2_ was passed through 0.22 μm pore size filters (Millipore Cat# SLGPR33RB, MA, USA) for sterilization and stored at 4 °C.

### In vitro calcium imaging

Seizure activity induced by 0-Mg^2+^ buffer in cultured hippocampal neurons is characterized by spontaneous repetitive intracellular calcium ([Ca^2+^]_i_) oscillations [66]. To confirm the successful induction of seizure activity in vitro, Fluo-4, AM (Solarbio Cat# F8501, Beijing, China) was used to monitor [Ca^2+^]_i_ oscillations. Fluo-4, AM stock solution of 5 mM was prepared in DMSO. Cultures grown in glass-bottom dishes were incubated with 2 μM Fluo-4, AM for 15 min at 37 °C. Then cultures were re-incubated with maintenance medium for another 30 min at 37 °C for the de-esterification of the dye. Cells were then transferred onto the recording chamber of an Olympus confocal inverted microscope (Olympus Confocal Laser Scanning Microscope Fluoview FV3000, RRID: SCR_017015, Tokyo, Japan). Fluorescence was excited with a 488 nm laser and emitted light was collected at 515 nm. A series of images were acquired every 1 s in a timeframe of 5 min at room temperature (RT). Cultures were changed to 0-Mg^2+^ buffer or control buffer and images were collected for a total of 1 h. Images were analysed using the cellSens Dimension software Desktop 3.1 (Olympus cellSens Software, RRID: SCR_014551, Tokyo, Japan). Cell bodies were selected as regions of interest and normalized fluorescence intensity changes were calculated as △*F/F* = (*F-F*_0_)/*F*_0_ (F, fluorescence intensity; F_0_, baseline intensity).

### Immunohistochemistry (IHC)

Rats were euthanized and perfused transcardially with normal saline followed by ice-cold 4% paraformaldehyde (PFA) for brain fixation. The brains were postfixed in 4% PFA for 2–4 h and transferred into 30% sucrose dissolved in 0.1 M PB solution for dehydration. Free-floating coronal brain sections of 30 μm were cut with a cryostat microtome (Leica CM1950 Cryostat, RRID:SCR_018061, Nussloch, Germany) and were stored in PBS containing 70% glycerol at −20 °C until use. As for the observation of the general distribution of NPCT throughout the rat brain, DAB-based immunohistochemistry using a commercial mouse streptavidin-horseradish peroxidase (HRP) kit (CWbio Cat# CW2069, Beijing, China) was performed on representative coronal sections at Bregma 4.68 mm, 3.72 mm, 2.76 mm, 1.92 mm, −0.36 mm, −1.20 mm, −2.16 mm, −2.40 mm, −3.60 mm, and −4.80 mm, according to the rat brain atlas by George Paxinos and Charles Watson [67]. Briefly, after quenching endogenous peroxidase and blocking, sections were incubated with primary antibody (mouse monoclonal anti-NPCT 1:100; Novus Cat# NB120-14817, RRID:AB_791678, CO, USA) diluted with PBS containing 10% goat serum and 0.3% Triton X-100 at 4 °C overnight, followed by sequential incubation with goat anti-mouse biotinylated IgG, streptavidin-HRP, and visualization with DAB working solution (CWbio Cat# CW0125, Beijing, China). Haematoxylin staining solution (Beyotime Cat# C0107, Beijing, China) was used to counter-stain nucleus. The expression level of NPCT was evaluated according to the proportion and intensity of positive cells as previously described [68]. The percentage of positive cells was classified into five scores: 4 (76–100%), 3 (51–75%), 2 (26–50%), 1 (1–25%), and 0 (0%). The staining intensity was categorized into four scores: 3 (strong), 2 (moderate), 1 (weak), 0 (no staining). The percentage and intensity scores were multiplied to obtain a total IHC score ranging from 0 to 12 corresponding to the expression level of NPCT. A total IHC score of >9 to ≤12, >6 to ≤9, >3 to ≤6, >0 to ≤3, and 0 was defined as high, medium-high, medium, low, and no expression, respectively. Negative controls were carried out by omitting the primary antibody. As for the detection of IgG leakage into the hippocampus, coronal sections were incubated with goat anti-rat biotinylated IgG (1:500; SinoBiological Cat# SSA012, Beijing, China) at 4 °C overnight, followed by streptavidin-HRP and DAB visualization as mentioned before.

As for immunofluorescent staining of brain sections, coronal brain sections were permeabilized and blocked with 0.3% Triton X-100 combined with 10% goat serum in PBS. The sections were incubated at 4 °C overnight with the following primary antibodies: mouse anti-NPCT (1:100; Novus Cat# NB120-14817, RRID:AB_791678, CO, USA), rabbit anti-NeuN (1:500; Millipore Cat# ABN78, RRID:AB_10807945, MA, USA), rabbit anti-GFAP (1:2000; Agilent Cat# Z0334, RRID:AB_10013382, CA, USA), rabbit anti-Iba1 (1:2000; FUJIFILM Wako Shibayagi Cat# 019-19741, RRID:AB_839504, Osaka, Japan), rabbit anti-PSD-95 (1:500; Abcam Cat# ab18258, RRID:AB_444362, MA, USA), and rabbit anti-synapsin (1:500; Abcam Cat# ab254349, RRID:AB_2920663, MA, USA). As for immunofluorescent staining of primary neurons, cells grown on cover glasses were fixed, permeabilized, blocked, and then incubated with the following primary antibodies at 4 °C overnight: rabbit anti-PSD-95 (1:500; Abcam Cat# ab18258, RRID:AB_444362, MA, USA), mouse anti-gephyrin (1:500; Synaptic Systems Cat# 147021, Goettingen, Germany), and mouse anti-MAP2 (1:500; Synaptic Systems Cat# 147021, RRID:AB_2232546, Goettingen, Germany). After washing, samples were incubated with the following secondary antibodies: goat anti-mouse IgG H&L [Alexa Fluor 488] (1:500; Thermo Fisher Scientific Cat# A-11001, RRID:AB_2534069, MA, USA), goat anti-rabbit IgG H&L [Alexa Fluor 594] (1:500; Thermo Fisher Scientific Cat# A-11005, RRID:AB_2534073, MA, USA) at RT for 2 h. Finally, nucleus was counter-stained with Hoechst (Solarbio C0021, Beijing, China) and samples were embedded with anti-fade mounting medium (SouthernBiotech Fluoromount-G Cat# 0100-01, AL, USA) and coverslips.

### Pre-absorption test

Pre-absorption test was carried out to examine the specificity of monoclonal anti-NPCT antibody used in the immunohistochemistry experiments and to confirm that this antibody could recognize NPCT without cross-reacting with PCT, the precursor peptide of NPCT. Dilutions of mouse monoclonal anti-NPCT antibody were incubated with NPCT peptide (2.5 μg NPCT for 1 μl undiluted antibody), or PCT peptide (5 μg PCT for 1 μl undiluted antibody; purified human PCT, Cat# hor-304, ProSpec, Rehovot, Israel), or without peptides, at 4 °C overnight. The dose of NPCT was chosen based on previous report where it could completely block staining [10]. The dose of PCT was calculated to obtain an approximate equimolar amount of peptide compared with NPCT, based on the molecular weight of NPCT (~8 kDa) and PCT (~13 kDa). This dose of PCT was also remarkably higher than that used in previous study [22], which ensured the effectiveness of PCT to block the monoclonal anti-NPCT antibody, if it could.

### Immunoelectron microscopy

Pre-embedding immunogold-silver method was performed as previously described [69] to investigate the subcellular distribution of NPCT in hippocampal pyramidal neurons. Briefly, rats were perfused with 150 ml normal saline followed by 500 ml ice-cold mixture of 4% PFA and 0.05% glutaraldehyde in 0.1 M PB for 1 h. Brains were removed and post-fixed in the same fixative at 4 °C for 4 h. Coronal sections of hippocampus of 45 μm were cut with a vibratome (Vibratome VT1000S Leica Microsystems, RRID:SCR_016495, Nussloch, Germany). Sections were blocked with 5% bovine serum albumin and 0.05% Triton X-100 in PBS for 3 h at RT, followed by incubation with the primary antibody of mouse anti-NPCT (1:100; Novus Cat# NB120-14817, RRID:AB_791678, CO, USA) overnight at RT and subsequent secondary antibody of anti-mouse IgG conjugated to 1.4 nm gold particles (1:100; Nanoprobes Cat# 2001, RRID:AB_2877644, NY, USA). After rinsing, sections were post-fixed in 2% glutaraldehyde in PBS for 45 min followed by silver enhancement performed in the dark with HQ Silver Kit (Nanoprobes Cat# 2012, NY, USA). Sections were rinsed with de-ionized water several times before and after silver enhancement. After being incubated with osmium tetroxide in 0.1 M PB for 2 h, sections were dehydrated with graded ethanol, then propylene oxide, and finally embedded in Epon 812 between plastic sheets. After polymerization, flat-embedded sections were examined under light microscope, trimmed, and glued onto blank resin stubs. Serial ultrathin sections were cut with an ultramicrotome (Leica EM UC6, Nussloch, Germany) with a diamond knife (Diatome, PA, USA), mounted on formvar-coated mesh grids (6-8 sections/grid), and then counterstained with uranyl acetate and lead citrate. Images were examined and captured with an electron microscope (JEOL 1230 Transmission Electron Microscope, RRID:SCR_018036, Tokyo, Japan) equipped with a CCD camera and its application software (Gatan 832 SC1000, CA, USA).

### FjC staining

FjC staining was used to detect degenerating neurons in hippocampus after RSE. Briefly, brain sections were mounted on slides and dried at 37 °C for 2 h. Then sections were sequentially immersed in 1% NaOH in 80% ethanol, 70% ethanol, distilled water, and 0.06% potassium permanganate solution for 5 min, 2 min, 2 min, and 20 min respectively. After rinsing with distilled water for 2 min, sections were incubated with 0.0001% FjC solution for 20 min. The 0.0001% FjC solution was prepared from the combination of 1 ml 0.01% FjC (Millipore Cat# AG325, MA, USA) stocking solution in distilled water and 99 ml 0.1% acetic acid. Sections were then rinsed and dried before mounting. Images were captured with a confocal microscope (Olympus Confocal Laser Scanning Microscope Fluoview FV3000, RRID:SCR_017015, Tokyo, Japan) with fluorescence excited with 488 nm laser.

### Lipofectamine transfection with GFP

In order to investigate the influence of NPCT on dendritic spine of primary hippocampal neurons, EGFP-plasmid (pEGFP-N1) was transfected to outline cell morphology as previously described [70]. Briefly, neurons of 13 DIV were transfected using Lipofectamine 2000 (Invitrogen 11668, CA, USA) for 4 h at 37 °C in 5% CO_2_/95% air. Each cover glass of neurons (in 24-well plate) was transfected with 1 μg DNA with the aid of 2 μl Lipofectamine 2000. Then neurons were washed and transferred back to their original medium with half changed with fresh medium and maintained at 37 °C, 5% CO_2_/95% air until 21 DIV. At 21 DIV, neurons were incubated with 1 nM of NPCT or d-NPCT for another 24 h at 37 °C, 5% CO_2_/95% air before imaging. Z-stack images of dendritic spine were acquired with a confocal microscope (Olympus Confocal Laser Scanning Microscope Fluoview FV3000, RRID:SCR_017015, Tokyo, Japan) with depth interval of 0.1 μm. The morphology of dendritic spine was 3D reconstructed based on z-stack image series with Imaris software v.9.5.0 (Imaris, RRID:SCR_007370, Abingdon, UK) and spine density was counted by an investigator blinded to the grouping.

### Sholl analysis of dendritic arborization

Primary hippocampal neurons at 5 DIV were incubated with 1 nM of NPCT or d-NPCT for 24 h and were subjected to anti-MAP2 immunofluorescent staining followed by sholl analysis to investigate the effects of NPCT on dendritic arborization. Sholl analysis was performed using Imaris software v.9.5.0 (Imaris, RRID:SCR_007370, Abingdon, UK) in steps of 10 μm starting from the soma. Both linear analysis and semi-log analysis were carried out according to previous study [71]. In linear sholl analysis, number of intersections with the circles (ranging from the soma with 10 μm steps) was plotted as a function of the radius of the circle. In the semi-log sholl analysis, the number of intersections within each circle area was calculated for each neuron, followed by the log value of this number being calculated. Then the log values were averaged from each neuron in a certain group and were plotted as a function of the distance from the soma. A straight line was then fitted to the data using simple linear regression. Sholl regression coefficient, k, was calculated by multiplying negative one with the slope of this fitting line for each group. The sholl regression coefficient accurately determines if two cell populations have different dendritic arborizations [72].

### Real-time quantitative PCR

Real-time quantitative PCR was conducted as previously described [73]. Total RNA of tissue and primary neurons was extracted with the aid of TRIzol reagent (Invitrogen Cat# 15596026, CA, USA) and then reverse-transcribed into cDNA using PrimeScript RT Master Mix (Takara Cat# RR036A, Kyoto Japan) according to the manufacturer’s instruction. A Bio-Rad analyser (Bio-Rad CFX96 Real-Time PCR Detection System, RRID:SCR_018064, CA, USA) was used for the real-time PCR analysis of cDNA. SYBR Green (bimake Cat# B21202, Shanghai, China) was used as the fluorescent probe. The gene encoding NPCT, *Calca*, could produce two transcripts, NPCT and CGRP, due to alternative splicing [74]. We designed three primer sets which were able to detect the common region of NPCT and CGRP (referred to as NPCT/CGRP), NPCT exclusively (referred to as NPCT), and CGRP exclusively (referred to CGRP), according to previous studies [16, 75]. All primers were synthesized by Tsingke Biotechnology (China) as follows: NPCT/CGRP, sense 5’-CCCTTTCCTGGTTGTCAGCATCTT-3’, antisense 5’-CATGCCTGGGCTGCTTTCTAAGGTT-3’; NPCT, sense 5’-CCCTTTCCTGGTTGTCAGCATCTT-3’, antisense 5’-AGCATGCAGGTACTCAGATTCCCA-3’; CGRP, sense 5’-AACCTTAGAAAGCAGCCCAGGCATG-3’, antisense 5’-GTGGGCACAAAGTTGTCCTTCACCA-3’; β-actin, sense 5’- ACGGTCAGGTCATCACTATCG-3’, antisense 5’-GGCATAGAGGTCTTTACGGATG-3’. Amplification conditions were as follows: one cycle of 95 °C for 3 min, 95 °C 5 s, 60 °C 30 s, 72 °C 30 s for additional 40 cycles. The relative mRNA expression of target gene was normalized to that of β-actin. Each experiment was repeated three times independently.

### Western-blot

The molecular weight of NPCT is lower than 10 kDa (~8 kDa) thus Tricine SDS-PAGE, which is more suitable to separate protein with low molecular weight, was performed to detect the expression of NPCT as previously described [76]. Molecules other than NPCT were separated with glycine-SDS-PAGE. Total cell and tissue protein was lysed with RIPA buffer (Beyotime Cat# P0013B, Beijing, China) supplemented with protease inhibitor (Roche Cat# 04693159001, Basel, Switzerland) and phosphatase inhibitor cocktail (Roche Cat# 04906837001, Basel, Switzerland). Each sample of 30 μg protein was run on 15.5% tricine-SDS-PAGE or 10% glycine-SDS-PAGE. Total proteins were transferred to polyvinylidene fluoride (Millipore Cat# ISEQ00010, MA, USA) membranes. After blocking with 5% non-fat milk in TBS with 0.1% Tween 20 for 1 h at RT, membranes were incubated at 4 °C overnight with primary antibodies including: mouse anti-NPCT (1:500; Novus Cat# NB120-14817, RRID:AB_791678, CO, USA), rabbit anti-caspase-3 (Proteintech Cat# 19677-1-AP, RRID:AB_10733244, Wuhan, China), rabbit anti-GAPDH (1:3000; HUABIO Cat# R1210-1, Hangzhou, China), and mouse anti-alpha tubulin (Proteintech Cat# 66031-1-Ig, RRID:AB_11042766, Wuhan, China). Goat anti-rabbit (1:5000; CWBio Cat# CW0103, RRID:AB_2814709, Beijing, China) and goat anti-mouse (1:5000; CWBio Cat# CW0102, RRID:AB_2814710, Beijing, China) HRP-conjugated secondary antibodies and enhanced chemiluminescence reagents (ZETA Cat# 310231, CA, USA) were applied to visualize interested protein bands. GAPDH and alpha tubulin were used as internal control.

### In vitro neuronal death assays

Neuronal death after in vitro SE model was assessed with PI/Hoechst double staining and phase-contrast morphological analysis at different time points as follows: immediately at the end of 0-Mg^2+^ exposure, and 3 h, 12 h, and 24 h after the end of 0-Mg^2+^ exposure. To investigate the influence of NPCT on neuronal survival after in vitro SE, 1 nM of NPCT or d-NPCT, or 50 μg/ml of Anti-NPCT or NRI were added into maintenance medium immediately after the end of 0-Mg^2+^ exposure and neuronal survival was investigated via PI/Hoechst double staining and phase-contrast morphological analysis 24 h later. PI/Hoechst double staining was performed with a commercial kit according to the manufacturer’s instruction (Solarbio Cat# CA1120, Beijing, China). PI is excluded from viable cells but stains dead cells following loss of membrane integrity and exhibits red fluorescence, whereas Hoechst 33342 labels all nuclei blue allowing the quantification of the total number of cells [77]. Fluorescence images were captured with a confocal microscope (Olympus Confocal Laser Scanning Microscope Fluoview FV3000, RRID:SCR_017015, Tokyo, Japan). The percentage of dead neurons (PI^+^ cells) to total cells (Hoechst^+^ cells) was calculated and the survival of neurons was expressed as 100% minus the percentage of dead neurons. Morphological identification of viable neurons was based on phase-contrast images as previously described [78]. Viable neurons were identified as phase-bright with intact processes while non-viable neurons were phase-dark and had fragmented processes. Survival of neurons was expressed as the percentage of the number of phase-bright cells to total number of cells.

### Cell Counting Kit-8 assay

Cell Counting Kit-8 (CCK-8) assay was used to assess the viability of primary hippocampal neuronal cultures according to the manufacturer’s instruction (DOJINDO Cat# CK04, Japan, Kumamoto, Japan). Cell viability was expressed as the percentage of the O.D. 450 nm relative to control levels.

### RNA-sequence and data analysis

Primary hippocampal neurons at 7 DIV were incubated with 1 nM NPCT or 1 nM PCT for 24 h and then subjected to RNA-sequence to interrogate the influences of NPCT and PCT on neuronal transcriptome. RNA-sequence was performed by Novogene co., ltd (Beijing, China). Briefly, total RNA was extracted from pooled samples of biological triplicates in each group using TRIzol reagent (Invitrogen Cat# 15596026, CA, USA) and was quantified and qualified with the Bioanalyzer 2100 system (Agilent Technologies, CA, USA; RRID:SCR_018043). Libraries were constructed, qualified, and sequenced by the Illumina NovaSeq 6000. Clean reads were aligned to the reference *Rattus norregicus* genome using Hisat2 (v2.0.5). The featureCounts v1.5.0-p3 was used to count the reads numbers mapped to each gene. The number of fragments per kilobase of transcript per million fragments mapped reads (FPKM) was then calculated and differential expression analysis between groups was performed using DESeq2 R package (1.20.0). The *P* values were adjusted using Benjamini and Hochberg’s approach for controlling the false discovery rate. Adjusted *P* value ≤ 0.05 and fold change > 1.5 was set as the threshold for significantly differential expression. Gene Ontology (GO) and Kyoto Encyclopaedia of Genes and Genomes (KEGG) enrichment analyses of differentially expressed genes (DEGs) were conducted with the clusterProfiler R package (3.8.1) [79]. All original RNA-sequence data have been deposited in GEO database with accession number: GSE217955.

### Seahorse XFe24 mitochondria respiration assay

To determine the overall effect of NPCT on mitochondrial respiration and OXPHOS, OCR was assessed using an Agilent Seahorse XFe24 Analyzer (Seahorse Biosciences, MA, USA; RRID:SCR_019539) [80]. Primary hippocampal neurons were seeded at 5 × 10^4^/well onto XFe24 plates and were maintained until 7 DIV before use. After stimulated with 1 nM NPCT for 24 h, cells were switched into XFe assay buffer at 37 °C without CO_2_ for 1 h. Following three baseline measurements of OCR, oligomycin (1 μM), FCCP (1 μM), and rotenone (1 μM) with antimycin A (10 μM) were sequentially injected into each well. Three OCR readings were taken after each injection. Basal respiration was calculated as OCR before oligomycin minus OCR after antimycin. Maximal respiration was calculated as OCR after FCCP minus OCR after antimycin. Spare respiratory capacity% was calculated as the percentage of maximal respiration to basal respiration. Proton leak was calculated as OCR after oligomycin minus OCR after antimycin. Data were normalized to cell number of each well.

### ATP assay

ATP levels of rat hippocampal tissue and primary hippocampal neurons were measured according to the instructions of a commercially available kit (Solarbio Cat# BC0300, Beijing, China). Absorbance at 340 nm was measured with a spectrophotometer (Tecan Infinite M200 Pro, RRID:SCR_019033, Crailsheim, Germany). Samples of rat hippocampus were collected at 2 h after DZP administration. Samples of primary hippocampal neurons were collected after neurons of 7 DIV were stimulated with 1 nM NPCT or d-NPCT for another 24 h.

### Mitochondria respiratory chain complex activity assays

The activities of mitochondria respiratory chain complexes I-V were measured according to instructions of commercial assay kits (complex I, Solarbio Cat# BC0515, Beijing, China; complex II, Solarbio Cat# BC3235, Beijing, China; complex III, Solarbio Cat# BC3245, Beijing, China; complex IV, Solarbio Cat# BC0945, Beijing, China; complex V, Solarbio Cat# BC1445, Beijing, China). Briefly, after neurons of 7 DIV were incubated with 1 nM NPCT or d-NPCT for another 24 h, samples of primary hippocampal neurons were homogenized with mitochondria isolation buffer and centrifuged at 600 g for 10 min at 4 °C. Supernatant was transferred to a new tube and subsequently centrifuged at 11,000 g for 15 min at 4 °C. The supernatant was then discarded, and the pellet enriched with mitochondria was resuspended in appropriate buffer for mitochondria complex activity assessment. Complex I activity was quantified by monitoring the oxidation of NADH to NAD^+^. The rate of NADH oxidation was determined by the decrease in the absorbance at 340 nm. Complex II activity was evaluated by decrease in the absorbance at 605 nm. Complex III activity was assessed by the increase of absorbance value at 550 nm after reduction of cytochrome c. Complex IV activity was measured by monitoring the decrease in the absorbance value at 550 nm after oxidation of reduced cytochrome c. Complex V activity was assessed by the increase in the absorbance value at 660 nm.

### JC-1

The changes in MMP of primary hippocampal neurons were monitored using JC-1 staining, a dual emission probe that exists as monomers with green fluorescence at low MMP and forms aggregates with red fluorescence at high MMP [81]. Primary hippocampal neurons were seeded and maintained in glass bottom dishes until 7 DIV and were stimulated with 1 nM NPCT or d-NPCT for another 24 h. After washing with warm HBSS three times, JC-1 (1:200; Solarbio Cat# M8650, Beijing, China) was diluted with NB, added back to the dishes, and incubated for 20 min at 37 °C and 5% CO_2_/95% air. The change of red and green fluorescence was monitored with a confocal microscope (Olympus Confocal Laser Scanning Microscope Fluoview FV3000, RRID:SCR_017015, Tokyo, Japan). The ratio of red/green fluorescence intensity was analysed using ImageJ software v.1.52a (National Institutes of Health, MD, USA; RRID:SCR_003070). The alterations of MMP in the in vitro SE model was monitored by the same JC-1 staining method, at the time points as those in the in vitro neuronal death assays.

### Statistical analysis

Statistical analyses were performed using GraphPad Prism v.9.0 (GraphPad Software, CA, USA; RRID:SCR_002798) and SPSS v.25 (IBM Corporation, NY, USA; SPSS, RRID:SCR_002865). Band intensities of western-blot, and intensities of IgG extravasation were analysed with ImageJ software v.1.52a (National Institutes of Health, MD, USA; RRID:SCR_003070). Fluorescent images captured with same parameters were analysed with Image Pro Plus v.6.0 (Media Cybernetics, MD, USA; RRID:SCR_016879) and ImageJ software v.1.52a (National Institutes of Health, MD, USA; RRID:SCR_003070). Experiments were repeated independently at least three times. Shapiro–Wilk test was used to determine the normality of data. *F* test (2 groups) and Brown–Forsythe test (3 or more groups) were used to test the homogeneity of variance. Continuous variables were presented as mean ± SEM for normal distribution and median with interquartile range (IQR) for skewed distribution. Student’s t-test (2 groups), one-way ANOVA with Turkey’s or Dunnett’s post-hoc analyses (3 groups and more) were used to compare data with normal distribution, and Welch’s correction (with Dunnett’s T3 post-hoc analyses for multiple comparison of 3 or more groups) was performed if variances were significantly different. Mann–Whitney *U* test (2 groups) and Kruskal–Wallis *H* test with Dunn’s post hoc analyses (3 groups and more) were used to compare data with skewed distribution. Repeated-measure two-way ANOVA or Mixed effects model with Turkey’s post-hoc analyses were used to assess statistical difference over time. Categorical data were presented as numbers with percentages and were compared with Chi-square test. Log-rank test was used to compare survival over time. Investigators who analysed EEG data were blinded to grouping allocation. Investigators in other experiments (such as western blots, real-time PCR, etc.) were unblinded. Two-sided *P* < 0.05 was considered statistical significance.

## Supplementary information

Supplementary Figures

Uncropped western blots

aj-checklist

## Data Availability

The datasets generated during and/or analysed during the current study are available from the corresponding author on reasonable request.
