# Supplementary material for: Aminoprocalcitonin protects against hippocampal neuronal death via preserving oxidative phosphorylation in refractory status epilepticus
Source: Cell Death Discov. 2023 May 4;9:144. doi: 10.1038/s41420-023-01445-7 (PMC10160063; doi:10.1038/s41420-023-01445-7)
Supplement: Supplementary file 1 — Supplementary Figures [file 41420_2023_1445_MOESM1_ESM.pdf]

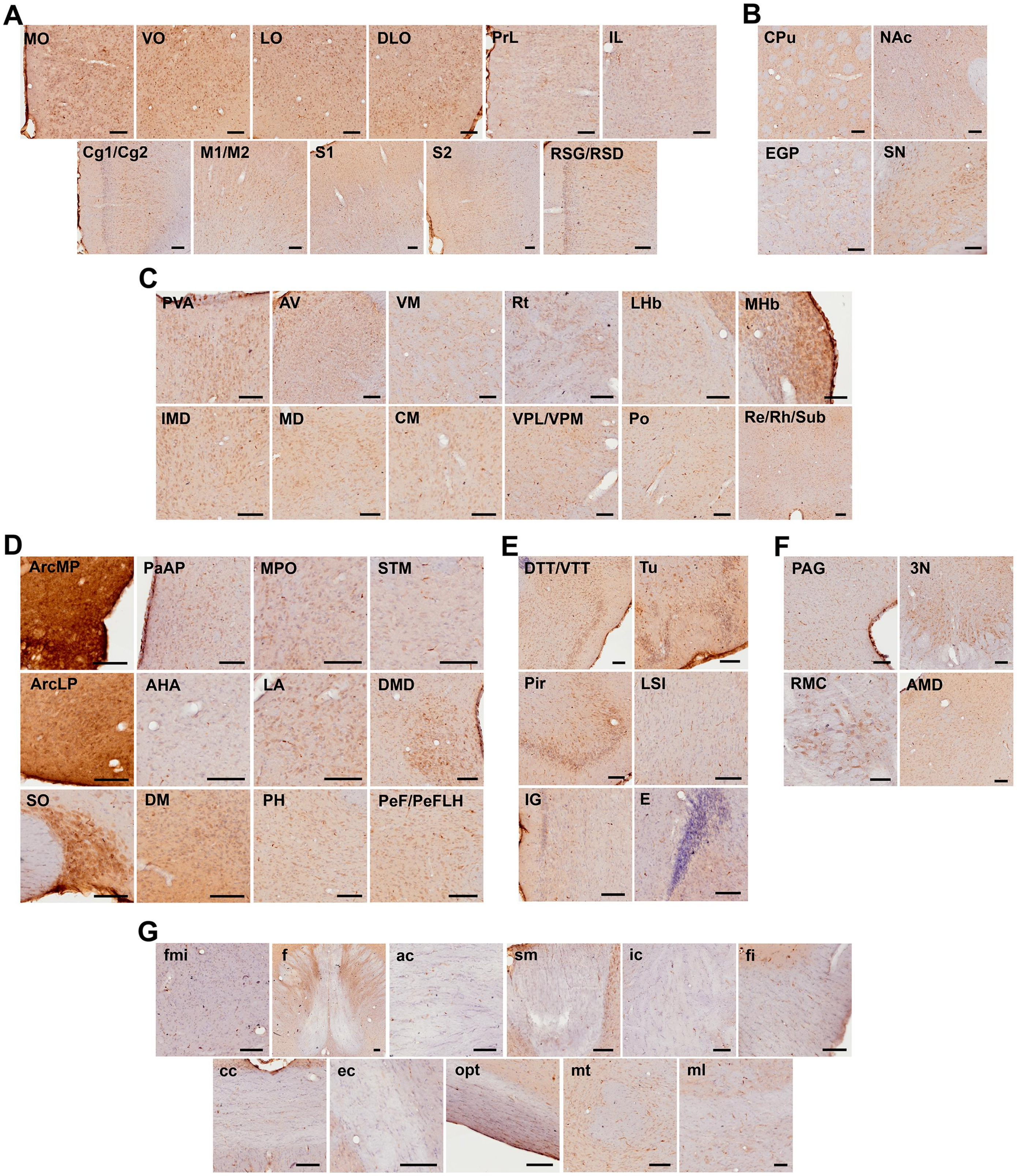


**Figure S1. NPCT is extensively distributed throughout the grey matter in the rat brain. (A)** The distribution of NPCT in subfields of the cerebral cortex. **(B)** The distribution of NPCT in striatal structures including the caudate putamen, nucleus accumbens, and the external globus pallidus, as well as its distribution in substantia nigra. **(C)** The distribution of NPCT in thalamic nuclei. **(D)** The distribution of NPCT in hypothalamic nuclei. **(E)** The distribution of NPCT in other representative brain regions. **(F)** The distribution of NPCT in midbrain nuclei and amygdala. **(G)** The distribution of NPCT in nerve structures. Bar scale = 100 μm in all images. Abbreviations: 3N: oculomotor nucleus; ac: anterior commissure; ArcLP: arcuate hypothalamic nucleus, lateroposterior part; ArcMP: arcuate hypothalamic nucleus, medial posterior part; AHA: anterior hypothalamic area, anterior part; AMD: amygdala; AV: anteroventral thalamic nucleus; cc: corpus callosum; Cg1/Cg2: cingulate cortex area 1/2; CM: central medial thalamic nucleus; CPu: caudate putamen; DLO: dorsolateral orbital cortex; DM: dorsomedial hypothalamic nucleus; DMD: dorsomedial hypothalamic nucleus, dorsal part; DTT/VTT: dorsal tenia tecta/ventral tenia tecta; E: ependyma and

subependymal layer; ec: external capsule; EGP: external globus pallidus; f: fornix; fi: fimbria of the hippocampus; fmi: forceps minor of the corpus callosum; ic: internal capsule; IG: indusium griseum; IL: infralimbic cortex; IMD: intermediodorsal thalamic nucleus; LA: lateroanterior hypothalamic nucleus; LHb: lateral habenular nucleus; LO: lateral orbital cortex; LSI: lateral septal nucleus, intermediate part; M1/M2: primary/secondary motor cortex; MD: mediodorsal thalamic nucleus; ml: medial lemniscus; MHb: medial habenular nucleus; MO: medial orbital cortex; MPO: medial preoptic nucleus; mt: mammillothalamic tract; NAc: nucleus accumbens; opt: optic tract; PaAP: paraventricular hypothalamic nucleus, anterior parvicellular part; PAG: periaqueductal gray; PeF/PeFLH: perifornical nucleus/perifornical part of lateral hypothalamus; PH: posterior hypothalamic nucleus; Pir: piriform cortex; Po: posterior thalamic nuclear group; PrL: prelimbic cortex; PVA: paraventricular thalamic nucleus, anterior part; Re/Rh/Sub: reuniens thalamic nucleus/rhomboid thalamic nucleus/submedius thalamic nucleus; RMC: red nucleus, magnocellular part; RSG/RSD retrosplenial granular/dysgranular cortex; Rt: reticular thalamic nucleus; S1: primary somatosensory cortex; S2: secondary somatosensory cortex; sm: stria medullaris of the thalamus; SN: substantia nigra; SO: supraoptic nucleus; STM: bed nucleus of the stria terminalis, medial division; Tu: olfactory tubercle; VM: ventromedial thalamic nucleus; VO: ventral orbital cortex; VPL/VPM: ventral posterolateral/posteromedial thalamic nucleus.

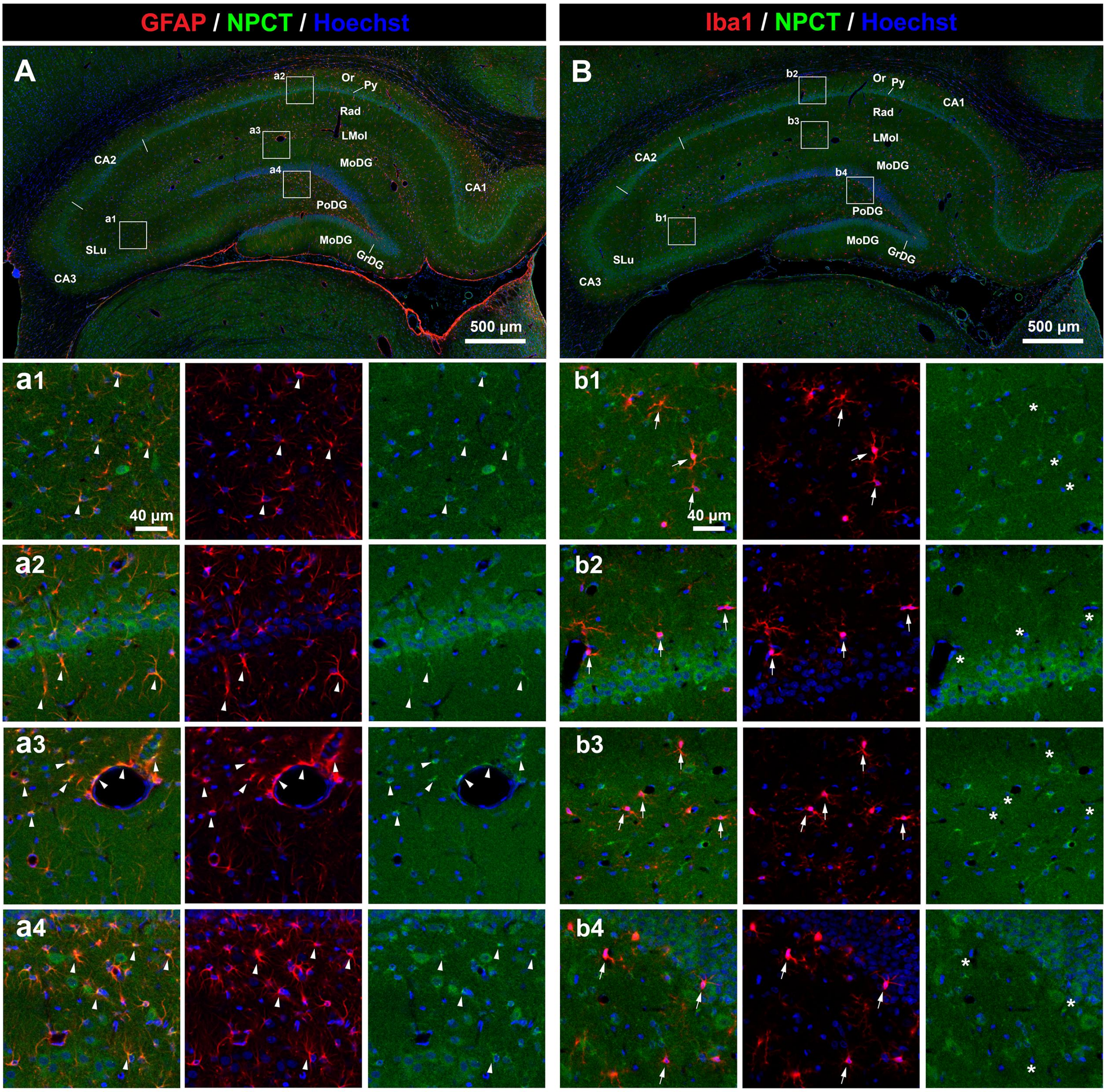


**Figure S2. The expression of NPCT in hippocampal astrocytes and microglia under physiological conditions. (A)** Panorama showing the colocalization of NPCT with GFAP, the marker of astrocytes, in different territories of the hippocampus. (a1-a4) Magnifications of insets from (A). Note that NPCT was concentrated within the somata of astrocytes (arrowheads). **(B)** Panorama showing the colocalization of NPCT with Iba1, the marker of microglia, in different territories of the hippocampus. (b1-b4) Magnifications of insets from (B) showing that NPCT (asterisks in the right column) was not expressed in microglia (arrows in the left and middle columns). n = 3 rats. Abbreviations: CA1: field CA1 of the hippocampus; CA2: field CA2 of the hippocampus; CA3: field CA3 of the hippocampus; GrDG: granular layer of the dentate gyrus; LMol: lacunosum moleculare layer of the hippocampus; MoDG: molecular layer of the dentate gyrus; Or: oriens layer of the hippocampus; PoDG: polymorph layer of the dentate gyrus; Py: pyramidal cell layer of the hippocampus; Rad: radiatum layer of the hippocampus; SLu: stratum lucidum of the hippocampus.

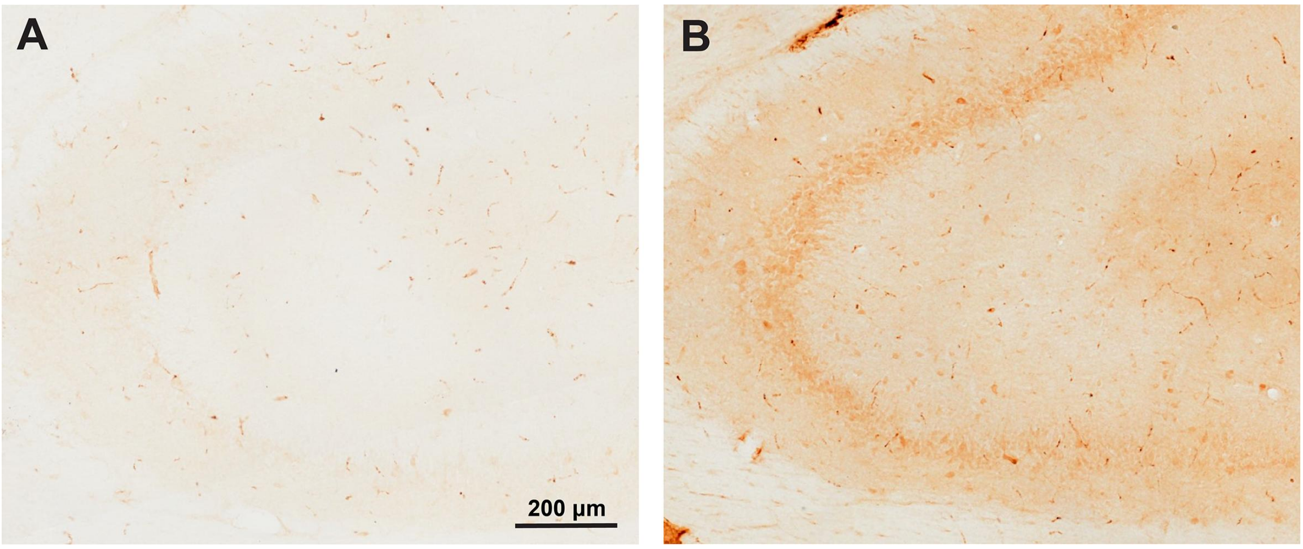


**Figure S3.** Negative control omitting NPCT primary antibody (**A**) shows no specific staining compared with staining with primary antibody (**B**).

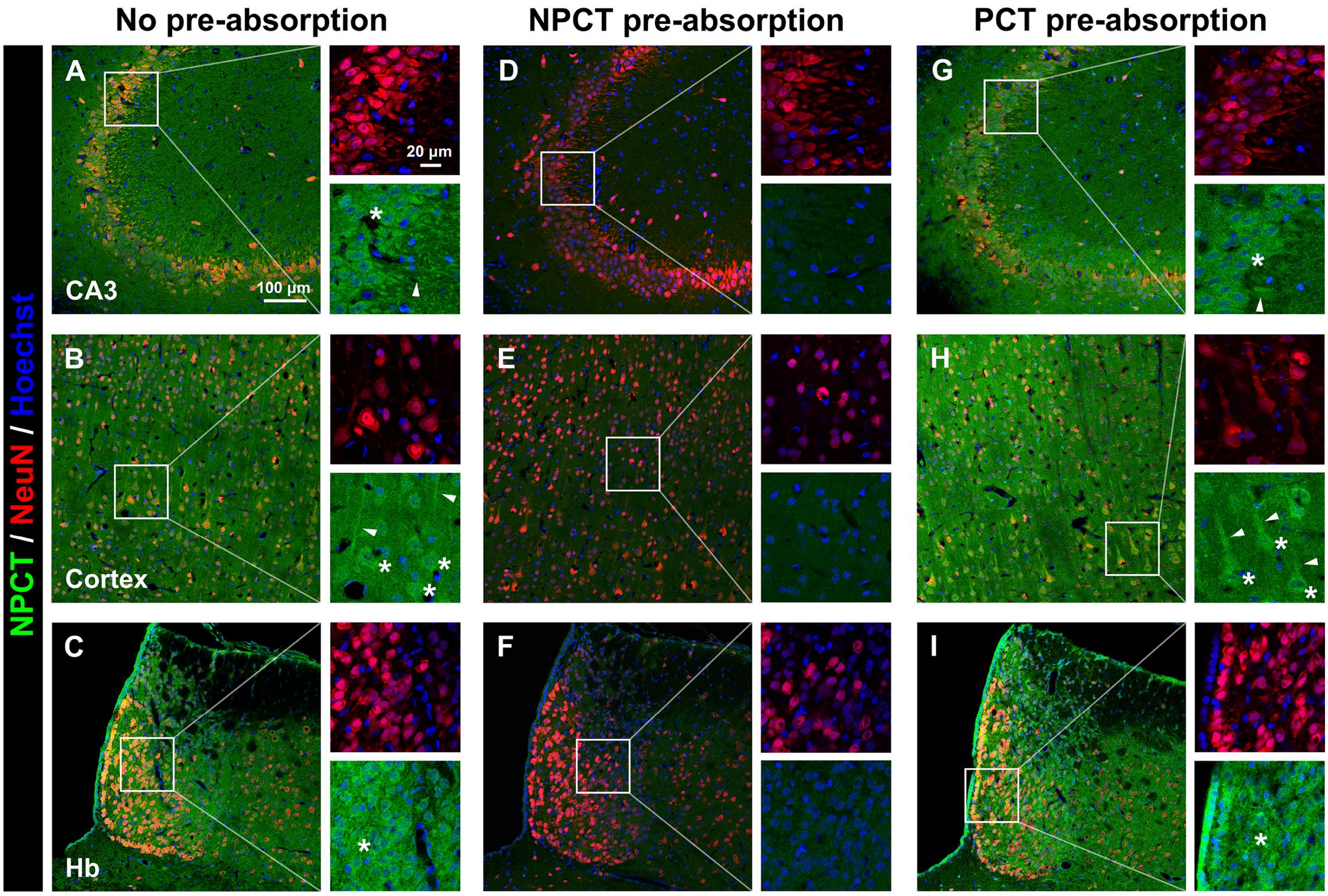


**Figure S4. Pre-absorption test demonstrating that NPCT, rather than the precursor PCT, could block the immunostaining of the monoclonal antibody specifically targeting NPCT. (A-C)** Immunofluorescent staining of NPCT using NPCT-specific monoclonal antibody without pre-absorption in CA3 of the hippocampus, cortex, and the Hb, counterstained with the neuronal marker NeuN (red) and nuclei (blue). Enriched NPCT fluorescent signals in neuronal somata (asterisks in the magnifications of insets) and dendrites (arrowheads in the magnifications of insets) could be noticed. **(D-F)** NPCT-specific monoclonal antibody was completely blocked by pre-absorption with NPCT peptide. No positive fluorescent signal could be detected in any of the brain region. **(G-I)** Pre-absorption with the parent peptide PCT could not block the NPCT-targeting monoclonal antibody. Enriched NPCT fluorescent signals in neuronal somata (asterisks in the magnifications of insets) and dendrites (arrowheads in the magnifications of insets) could still be clearly noticed. n = 3 independent experiments. Abbreviations: CA3: field CA3 of the hippocampus; Hb: habenula nucleus.

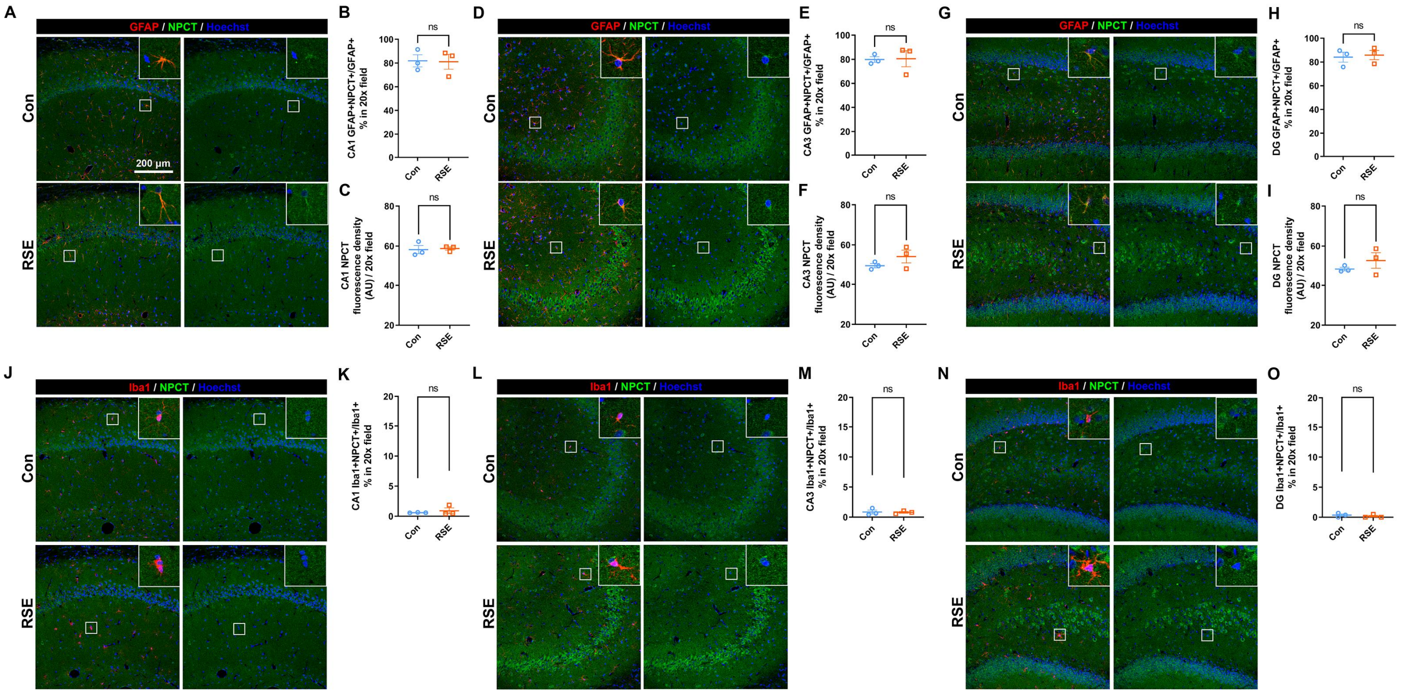


**Figure S5. The expression of NPCT in hippocampal astrocytes and microglia in RSE.** NPCT was co-stained with the markers of astrocyte (GFAP) and microglia (Iba1). **(A-C)** The expression of NPCT in CA1 astrocytes in RSE. One hour of RSE did not alter either the proportion of astrocytes that expressed NPCT (B) or NPCT fluorescent density in astrocytes (C). **(D-F)** The expression of NPCT in CA3 astrocytes in RSE. One hour of RSE did not alter either the proportion of astrocytes that expressed NPCT (E) or NPCT fluorescent density in astrocytes (F). **(G-I)** The expression of NPCT in DG astrocytes in RSE. One hour of RSE did not alter either the proportion of astrocytes that expressed NPCT (H) or NPCT fluorescent density in astrocytes (I). **(J-O)** The expression of NPCT in microglia in CA1 (J-K), CA3 (L-M), and DG (N-O). One hour of RSE did not alter the proportions of microglia that expressed NPCT in CA1 (K), CA3 (M), and DG (O). Considering that NPCT was not colocalized with microglia as shown in (J, L, N), the NPCT fluorescent density within microglia was not investigated. Student's t test. n = 3 rats in each group. All data are presented as mean ± SEM.

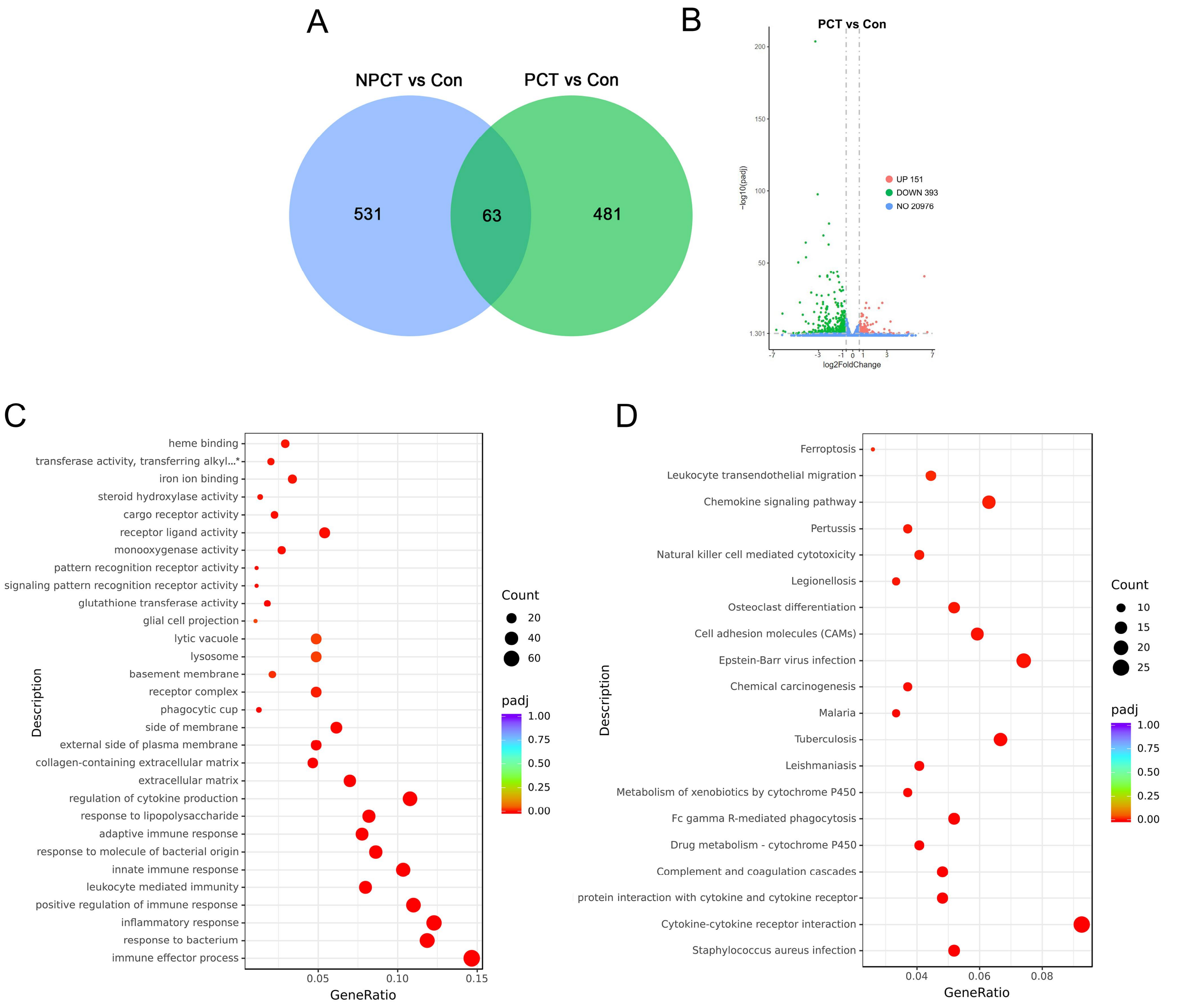


**Figure S6. Transcriptome alterations of primary hippocampal neurons after PCT stimulation. (A)** The Venn diagram demonstrating the shared and unique differentially expressed genes (DEGs) of primary hippocampal neurons stimulated with 1 nM NPCT and 1 nM PCT for 24 h. **(B)** Volcano plots showing DEGs in PCT-stimulated primary hippocampal neurons. **(C)** GO enrichment analysis of DEGs from PCT-stimulated primary hippocampal neurons. Asterisk represents "transferase activity, transferring alkyl or aryl (other than methyl) groups". **(D)** KEGG enrichment analysis of DEGs from PCT-stimulated primary hippocampal neurons. n = 3 biological replicates.

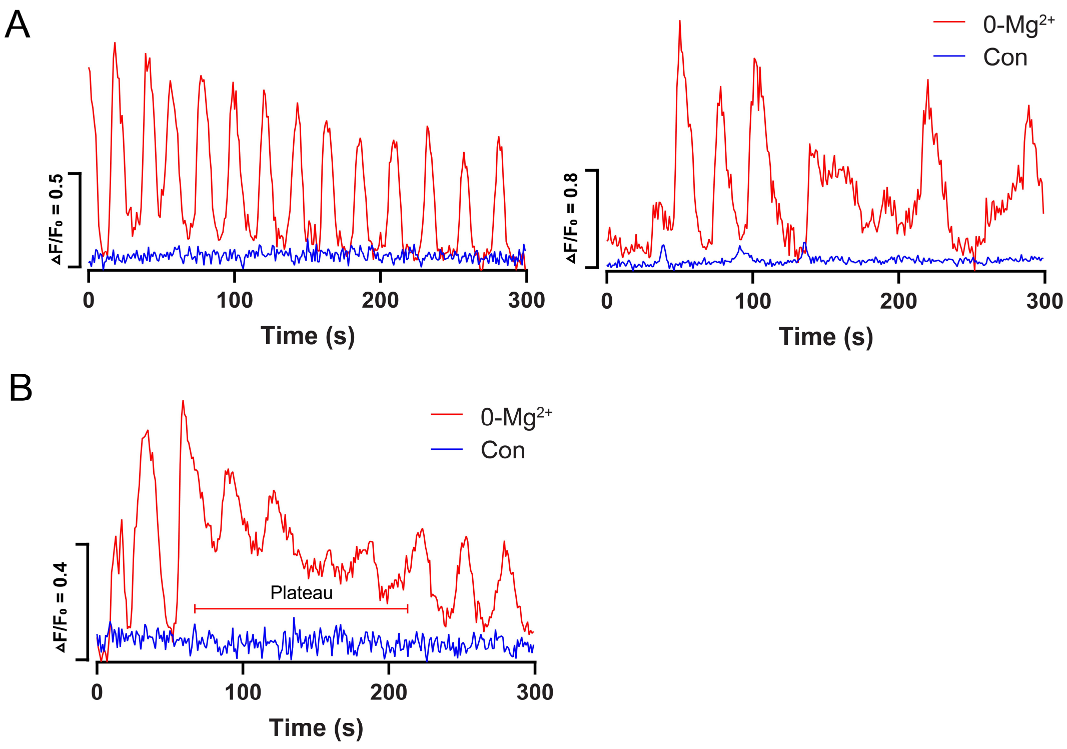


**Figure S7. (A-B)** Representative oscillations in $[Ca^{2+}]_i$ in individual primary hippocampal neuron induced by perfusion of 0-$Mg^{2+}$ buffer. Note that $[Ca^{2+}]_i$ oscillations sometimes fused to produce a plateau-like increase in $[Ca^{2+}]_i$ as denoted in (B).

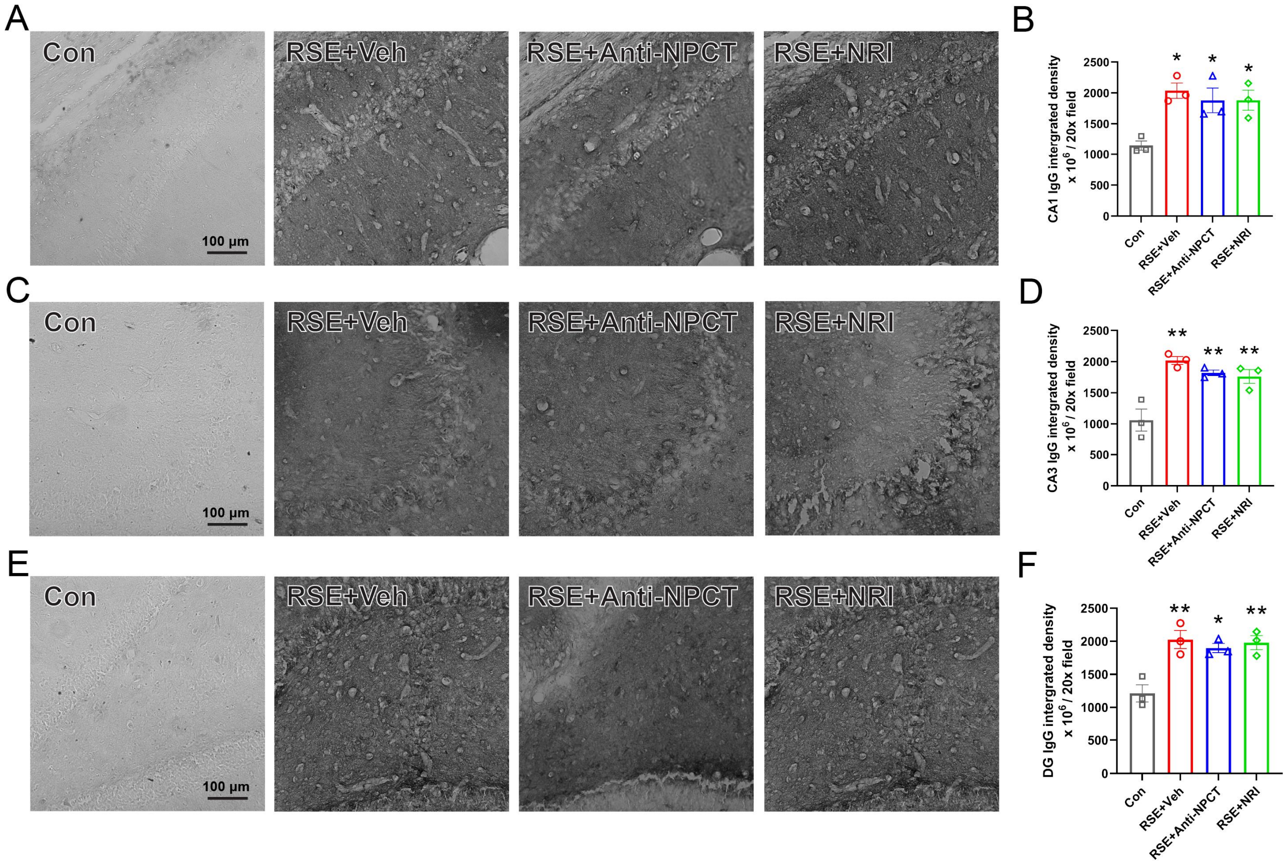


**Figure S8. RSE induced blood-brain barrier leakage. (A-B)** Immunohistochemistry staining of rat IgG in CA1 in control rats and RSE rats with intraperitoneal (i.p.) administration of vehicle (Veh), anti-NPCT, and nonimmune rabbit IgG (NRI). One-way ANOVA with Tukey's post hoc analyses. n = 3 rats in each group. *$P < 0.05$, compared with control group. **(C-D)** Immunohistochemistry staining of rat IgG in CA3 in control rats and RSE rats with i.p. administration of Veh, anti-NPCT, and NRI. One-way ANOVA with Tukey's post hoc analyses. n = 3 rats in each group. **$P < 0.01$, compared with control group. **(E-F)** Immunohistochemistry staining of rat IgG in DG in control rats and RSE rats with i.p. administration of Veh, anti-NPCT, and NRI. One-way ANOVA with Tukey's post hoc analyses. n = 3 rats in each group. *$P < 0.05$, **$P < 0.01$, compared with control group. All data are presented as mean ± SEM.

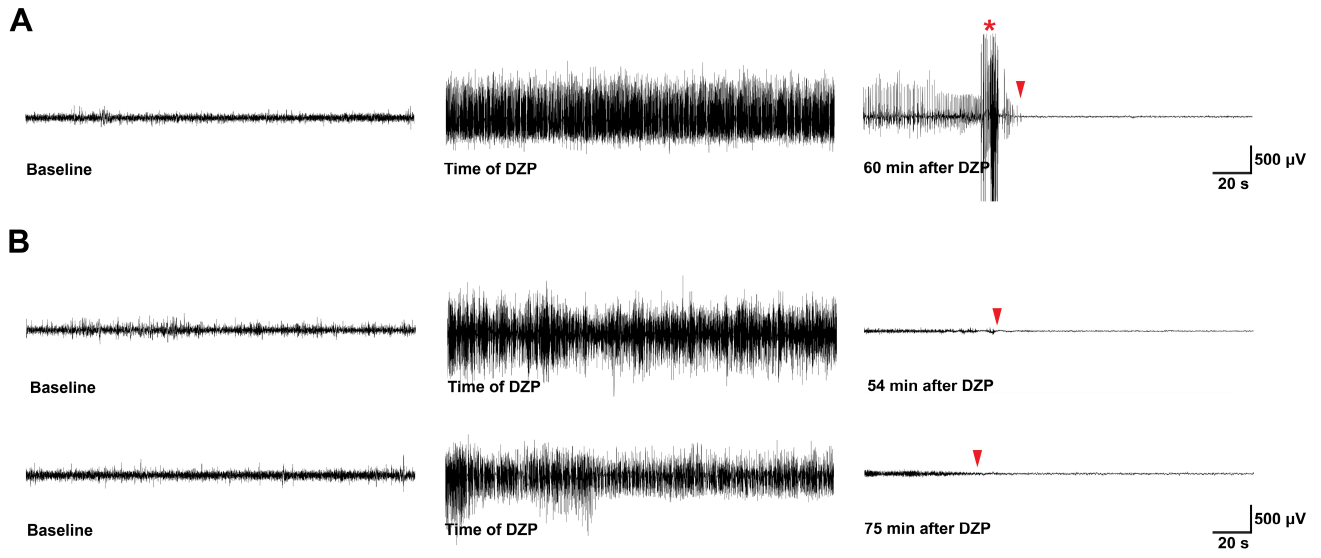


**Figure S9. Hippocampal EEG traces of animals that died during the video-EEG recording. (A)** An animal from the RSE+Veh group died following a series of violent generalized tonic-clonic seizures (asterisk in A) at 60 min after diazepam (DZP) administration. Arrowhead denotes the time when the animal died after which the EEG traces became flattened without any activity. **(B)** Two animals from the RSE+Anit-NPCT group died at 54 min (upper) and 75 min (lower) after DZP administration. Note that these two animals gradually died before which EEG traces had already exhausted and been terminated by DZP. Arrowheads denote the time when the animals died after which the EEG traces became completely flattened without any activity.
