# Supplementary figures and images for: Aminoprocalcitonin protects against hippocampal neuronal death via preserving oxidative phosphorylation in refractory status epilepticus
Source: Cell Death Discov. 2023 May 4;9:144. doi: 10.1038/s41420-023-01445-7 (PMC10160063; doi:10.1038/s41420-023-01445-7)

Uncropped western blots

Figure 2I

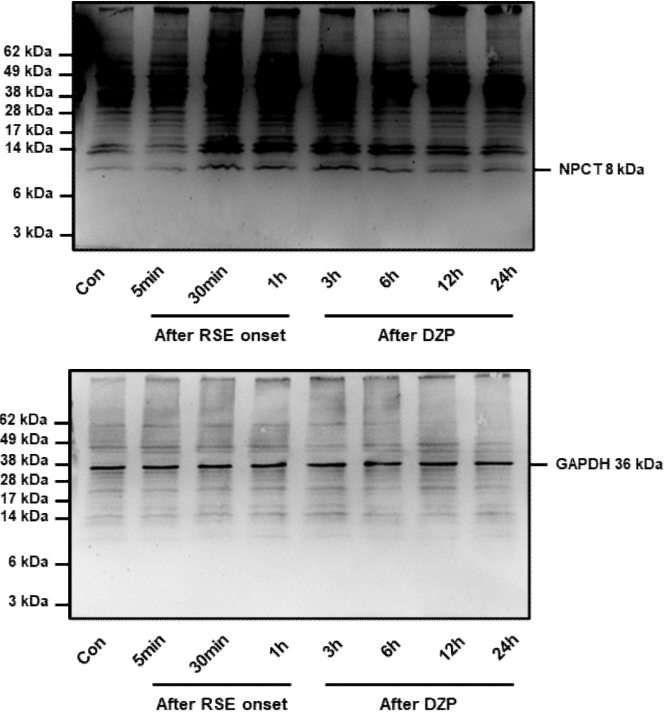

Figure 4M

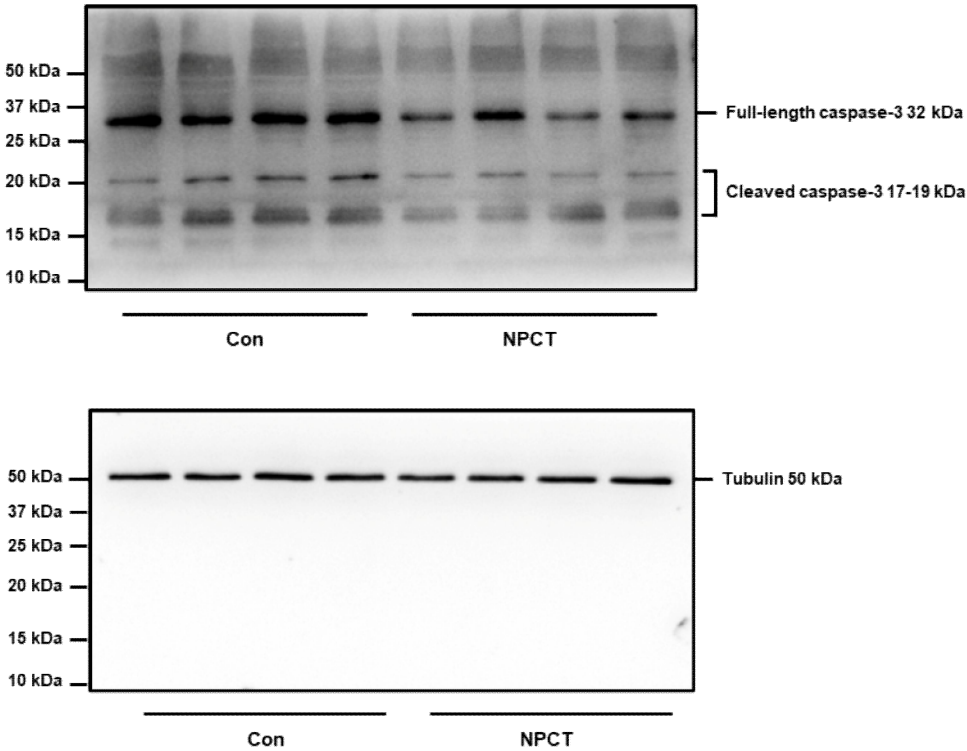

Supplement: Supplementary file 2 — Uncropped western blots [file 41420_2023_1445_MOESM2_ESM.pdf]
